# Meta-analysis of the histopathological features of surgically removed aortas classified according to the SCVP and AECVP consensus documents

**DOI:** 10.1016/j.ahjo.2026.100904

**Published:** 2026-09-25

**Authors:** Eemeli Jämsen, David Kalfeřt, Tero Halmesmäki, Ari Mennander, Timo Paavonen, Ivana Kholová

**Affiliations:** a Faculty of Medicine and Health Technology, Tampere University, Arvo Ylpön katu 34, 33520, Tampere, Finland; b Department of Otorhinolaryngology Head and Neck Surgery, First Faculty of Medicine, University Hospital Motol and Homolka, Charles University, V Uvalu 84, 15006, Prague, Czech Republic; c Division of Cardiothoracic Surgery, Tampere University Heart Hospital, Elämänaukio 1, 33520, Tampere, Finland; d Department of Pathology, Fimlab Laboratories, Arvo Ylpön katu 4, 33520, Tampere, Finland

**Keywords:** Aortic aneurysm, Aortic dissection, Bicuspid aortic valve, Consensus document, Histopathology, Medial degeneration, Meta-analysis

## Abstract

The Society for Cardiovascular Pathology (SCVP) and the Association for European Cardiovascular Pathology (AECVP) published consensus statements on the surgical pathology of the aorta to unify nomenclature and provide a new classification scheme to systematically evaluate the degenerative and inflammatory lesions of the thoracic aorta. The aim of this meta-analysis was to determine the histopathological features of thoracic aneurysmatic aortas, thoracic dissected aortas, and thoracic aortas in patients with a bicuspid aortic valve (BAV). Additionally, the aim was to map how consensus documents and classification schemes is used in practice and research.

Articles for analysis were identified while searching for references to the SCVP and AECVP consensus documents, resulting in 174 articles. After applying the exclusion and inclusion criteria, 10 articles were included in the analysis. Four cohorts were analysed: the whole cohort, the aneurysm cohort, the dissection cohort, and the BAV cohort.

Medial degeneration was found in all cohorts. The dissection cohort showed more severe medial degeneration than the aneurysm cohort or the BAV cohort. The BAV cohort showed the mildest medial degeneration compared to the other cohorts. More prevalent smooth muscle cell nuclei loss was linked to dissection. The BAV cohort showed milder changes in elastic fibers. Atherosclerosis was comparable among all cohorts.

Medial degeneration represents the pathological process of several aortic diseases. All studied components of medial degeneration were present in aneurysms, dissections, and BAV aortas. The consensus documents have been useful tools and assist with the systematic evaluation of resected aortas, although their use in the complexity is limited.

## Highlights

• Medial degeneration represents the pathological process in aortic disease.
• All studied components of medial degeneration were present in all cohorts.
• Dissected aortas show more severe MD compared to aneurysmatic and BAV aortas.
• Patients with BAV showed less severe medial degeneration.
• Atherosclerosis was comparable among all cohorts.

## Graphical abstract

**Figure:**
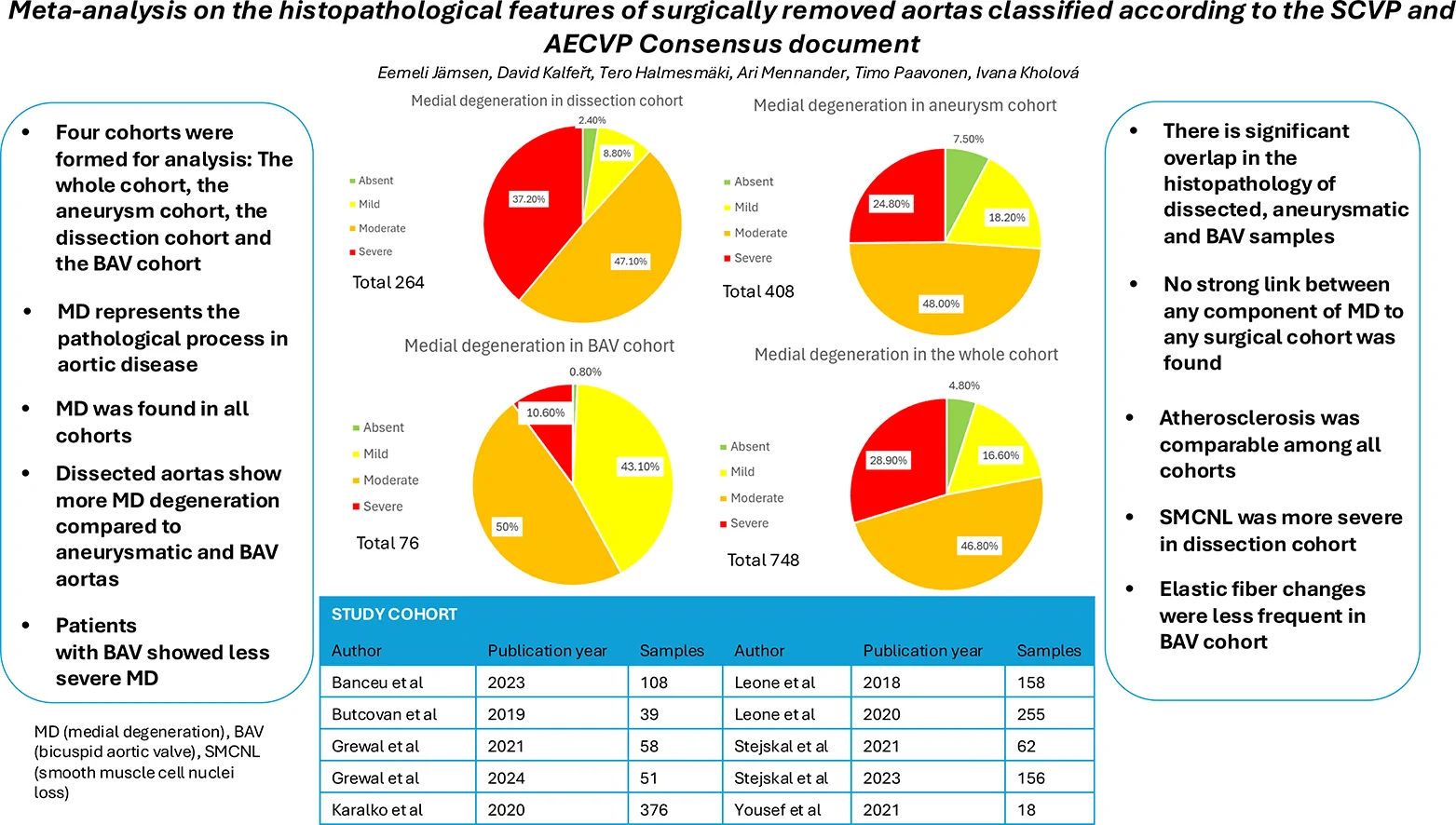

## 1 Introduction

Diseases of the thoracic aorta include aortic dilatation, aneurysm, and dissection. Some dilatated or aneurysmatic aortas may remain asymptomatic until an acute aortic event—including aortic dissection and rupture—occurs and prompt surgery is required. Understanding the underlying causes of these possibly lethal conditions enables healthcare professionals to deliver properly timed and cost-effective treatments that reduce morbidity and mortality and improve patients' quality of life [1].

Classically aortic dilatation is defined as an aortic diameter larger than two standard deviations of the predicted one when adjusted for age, sex and body size. Over time aortic dilatation can grow into an aortic aneurysm. An *aortic aneurysm* is defined as an aortic diameter more than 1.5 times the normal diameter. This definition has been contested, and recently more precise methods of risk stratification using a Bayesian model have been proposed [2], [3]. Nevertheless, the classical definition of aortic aneurysm is still widely used. The 2024 European Society of Cardiology Guidelines for the Management of Peripheral Arterial and Aortic Diseases estimate the prevalence of thoracic aortic aneurysm as 5–10/100,000 person years [2]. In thoracic aorta dissection (TAD), the innermost layer, the intima, tears and blood flow forms a false lumen in the medial layer of the aorta [2]. The prevalence of TAD is estimated to be 2.6–3.5/100,000 person years [2]. The risk factors are mostly shared among other arterial diseases, primarily male sex, age, and hypertension [1], [2].

An acute aortic event necessitates acute surgery in which the damaged part of aortic tissue is removed [2]. These resected aortas contain vital information about the structure of the aortic wall and the underlying processes that make them vulnerable. Therefore, it is recommended to collect these samples for histopathological analysis [4]. In 2015 and 2016, the Society for Cardiovascular Pathology (SCVP) and the Association for European Cardiovascular Pathology (AECVP) published consensus statements on the surgical pathology of the aorta. The first part covered inflammatory diseases of the aorta, and the second focused on non-inflammatory degenerative diseases [4], [5]. The purpose of the consensus documents was to unify the nomenclature, which had varied over time and led to inconsistencies. The purpose was also to provide a new classification scheme to more consistently evaluate degenerative lesions [5]. Unified terms and standard methods to describe changes in histopathology help promote a better understanding of the diseases affecting the aorta and make future research more accurate.

The classification scheme uses medial degeneration (MD) as a top-line term and is graded as none–mild–moderate–severe. MD is the sum of individual degenerative lesions. These individual components are mucoid extracellular matrix accumulation (MEMA) divided into intralamellar (MEMA-I) and translamellar (MEMA-T), elastic fiber fragmentation and/or loss (EFF/L), elastic fiber thinning (EFT), elastic fiber disorganization (EFD), smooth muscle cell nuclei loss (SMCNL), smooth muscle cell disorganization (SMCD), laminar medial collapse (LMC), and medial fibrosis (MF) divided into intralamellar (MF-I) and translamellar (MF-T). Thus far, no individual component has been strongly linked to any specific syndrome or surgical phenomenon affecting the aorta [6], [7], [8], [9].

In the present meta-analysis, we aimed to determine the complex histopathological features of thoracic aneurysmatic aortas, thoracic dissected aortas, and thoracic aorta samples from patients with bicuspid aortic valves in the context of the new classification scheme. Additionally, we aimed to map how the scheme is used in the published literature and, thus, the surgical pathology diagnostic workup of aortic specimens.

## 2 Materials and methods

Scientific articles for analysis were identified by searching with PubMed search engine in the MEDLINE database for references to the following published guidelines: “Consensus statement on surgical pathology of the aorta from the Society for Cardiovascular Pathology and the Association for European Cardiovascular Pathology: I. Inflammatory diseases” [4] and “Consensus statement on surgical pathology of the aorta from the Society for Cardiovascular Pathology and the Association for European Cardiovascular Pathology: II. Noninflammatory degenerative diseases—nomenclature and diagnostic criteria” [5].

This search resulted in a total of 174 articles: 79 articles referred to Consensus Document I, and 95 articles referred to Consensus Document II, respectively. The search was conducted between March 1, 2023, and March 2, 2024. To be included in this analysis, articles had to be prospective or retrospective surgical pathology or autopsy cohorts and grade the medial degeneration of resected thoracic aortic samples strictly using consensus statements [4], [5]. Reviews, case reports, meta-analyses, non-English articles, and animal studies were excluded from this analysis. In addition, reports from parts of the aorta other than the thoracic were excluded.

Based on the titles and abstracts, 10 of 174 articles met the inclusion criteria. As the PubMed “cited by” search was not comprehensive, six more articles were included from the author's archive as allowed by PRISMA guidelines [10]. Those articles exist in the MEDLINE database and cite the consensus documents, combining 16 articles that were read completely. Out of these 16 articles, six were incompatible for analysis due to their method of presenting the results leaving 10 articles for analysis in the literature (Fig. 1, Table 1).

**Fig. 1:**
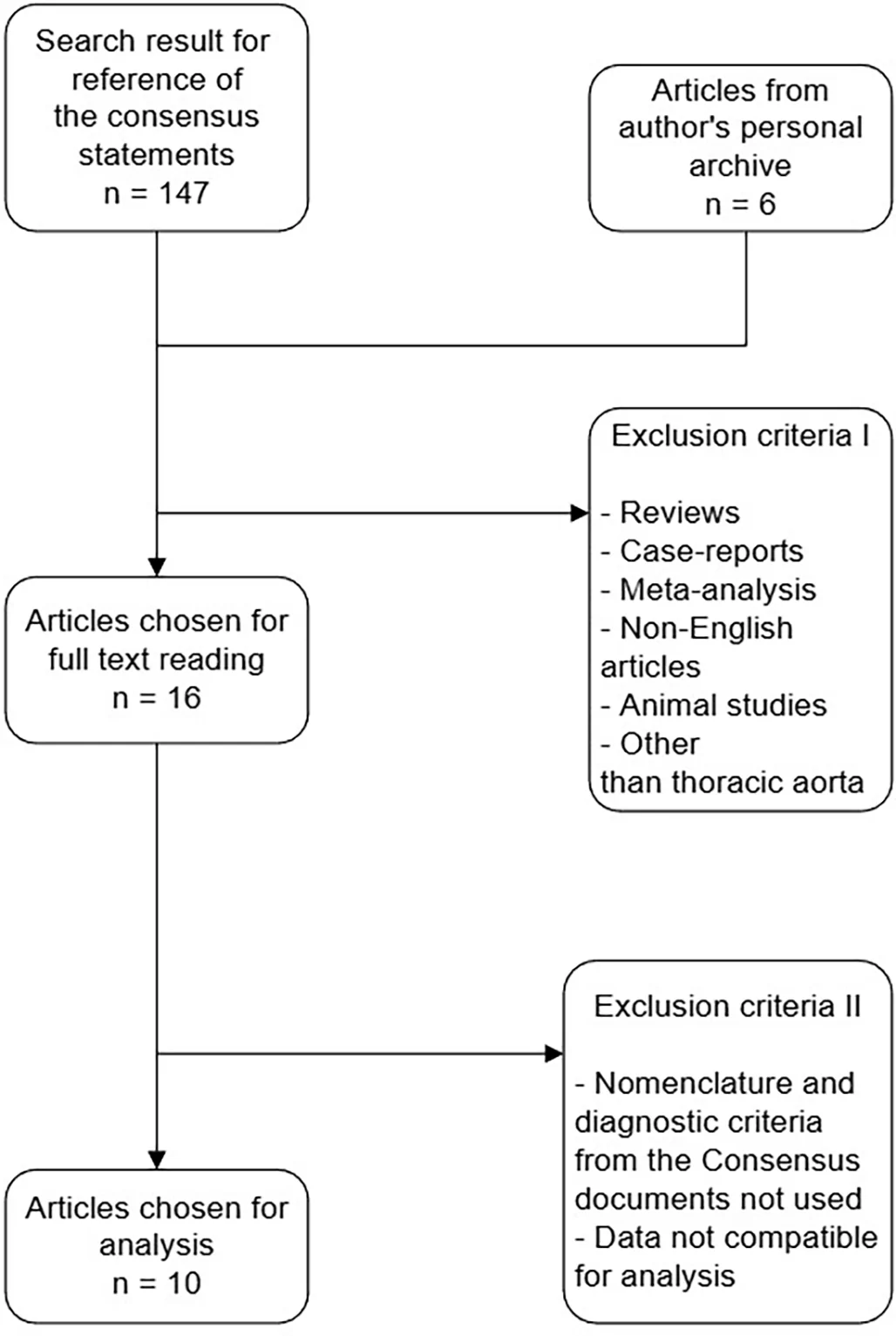
Flow chart describing the process of searching for and selecting studies for meta-analysis.

**Table 1:** Overview of the articles included in the current meta-analysis, n.d. not determined.

Authors	Publication year	Sample collection	PMID	Country	Aneurysm, n	Dissection, n	BAV, n
Banceu et al. [11]	2023	2019–2021	37895149	Romania	56	52	n.d.
Butcovan et al. [12]	2019	2015–2016	31572544	Romania	39	n.d.	n.d.
Grewal et al. [13]	2021	n.d.	33513898	The Netherlands	n.d.	58	n.d.
Grewal et al. [14]	2024	n.d.	38181821	The Netherlands	n.d.	51	n.d.
Karalko et al. [15]	2020	2009–2019	33332528	The Czech Republic	n.d.	n.d.	209
Leone et al. [16]	2018	2000–2013	29803371	Italy	n.d.	158	n.d.
Leone et al. [17]	2020	2015–2016	31706551	Italy	255	n.d.	n.d.
Stejskal et al. [18]	2021	2016–2020	34246045	The Czech Republic	n.d.	55	n.d.
Stejskal et al. [19]	2023	2018–2022	37172524	The Czech Republic	40	48	69
Yousef et al. [20]	2021	n.d.	34162971	USA	18	n.d.	n.d.

Microsoft Excel for Microsoft 365 MSO (Version 2111 Build 16.0.14701.20254) 64-bit was used to gather and compile data from the articles. The following information was collected: the first author, publication year, title, country, type of institution, sample collection period if disclosed, detailed characteristics of cohorts, and histological data of cohorts. Additionally, we collected indications for surgery and the removal of the resected aortas, as described in their original articles. Surgical indications, such as an aneurysm, dilatation, and dissection, were used for further categorization and analysis. Moreover, because some studies compared the histology of aortas between samples from patients with bicuspid aortic valves versus tricuspid aortic valves, a separate cohort of patients with bicuspid aortic valves was formed. Our meta-analysis followed the PRISMA 2020 checklist items [10].

All meta-analyses were performed using OpenMetaAnalyst (Brown University, Providence, RI, USA). For each study, the prevalence of individual histopathological features was calculated as the proportion of affected aortic specimens relative to the total number analysed. Pooled prevalence estimates were generated using a binary random-effects model with the DerSimonian–Laird method to account for between-study heterogeneity. A continuity correction of 0.5 was applied to the zero-event cells. The results are presented as proportions with 95% confidence intervals. Statistical heterogeneity was assessed using Cochran's Q test and quantified using the I^2^ statistic. Forest plots were used to visualize study-level and pooled estimates. A two-sided *p* < 0.05 was considered statistically significant.

## 3 Results

### 3.1 Cohort characteristics

The whole cohort included 10 studies and consisted of 1282 thoracic aortic samples. The average age was 61.0 SD ± 4.9 years, and males made up 69% of the samples (*n* = 897). In total, 378 samples from patients with bicuspid aortic valves (BAVs) were included. The aneurysm cohort included five studies with 408 samples. The average age was 61.3 SD ± 5.8 years. Males comprised 74.3% (*n* = 303) of the samples, and the aneurysm cohort included 91 samples with BAV. The dissection cohort consisted of six studies with 422 samples. The average age was 63.7 SD ± 1.1 years. Males made up 63.3% of the samples (*n* = 267), and the dissection cohort included 10 samples with BAV. The BAV cohort included only three studies with 284 samples. The average age was 54.7 SD ± 4.2 years, and 74.6% of the samples were from males (*n* = 212). The BAV cohort includes mostly dilatated samples with 277 of samples being dilatated and 7 dissected thoracic aorta samples.

At least 40 out of 408 samples in the aneurysm cohort were removed during elective procedures and 206 out of 422 dissected samples were removed during acute procedure. For the rest of the samples this information was not disclosed in the analysed publications.

### 3.2 Whole cohort

The most prevalent grade of overall medial degeneration in the whole cohort was moderate, with a prevalence of 46.8% (*n* = 358) out of 748 aortic samples across eight studies (95% CI 41.5%–52.0% with an inconsistency index of 45.47%). The second most common grade was severe, with a prevalence of 28.9% (*n* = 184; 95% CI 21.0–36.8%, I^2^ of 82.01%, *p* < 0.001). Only 16.6% (*n* = 157) samples were graded mild, and in 4.8% (*n* = 49) samples, medial degeneration was absent (Table 2; Fig. 2). Table 3 summarizes the most prevalent morphological features (Suppl. Fig. 1–10).

**Fig. 2:**
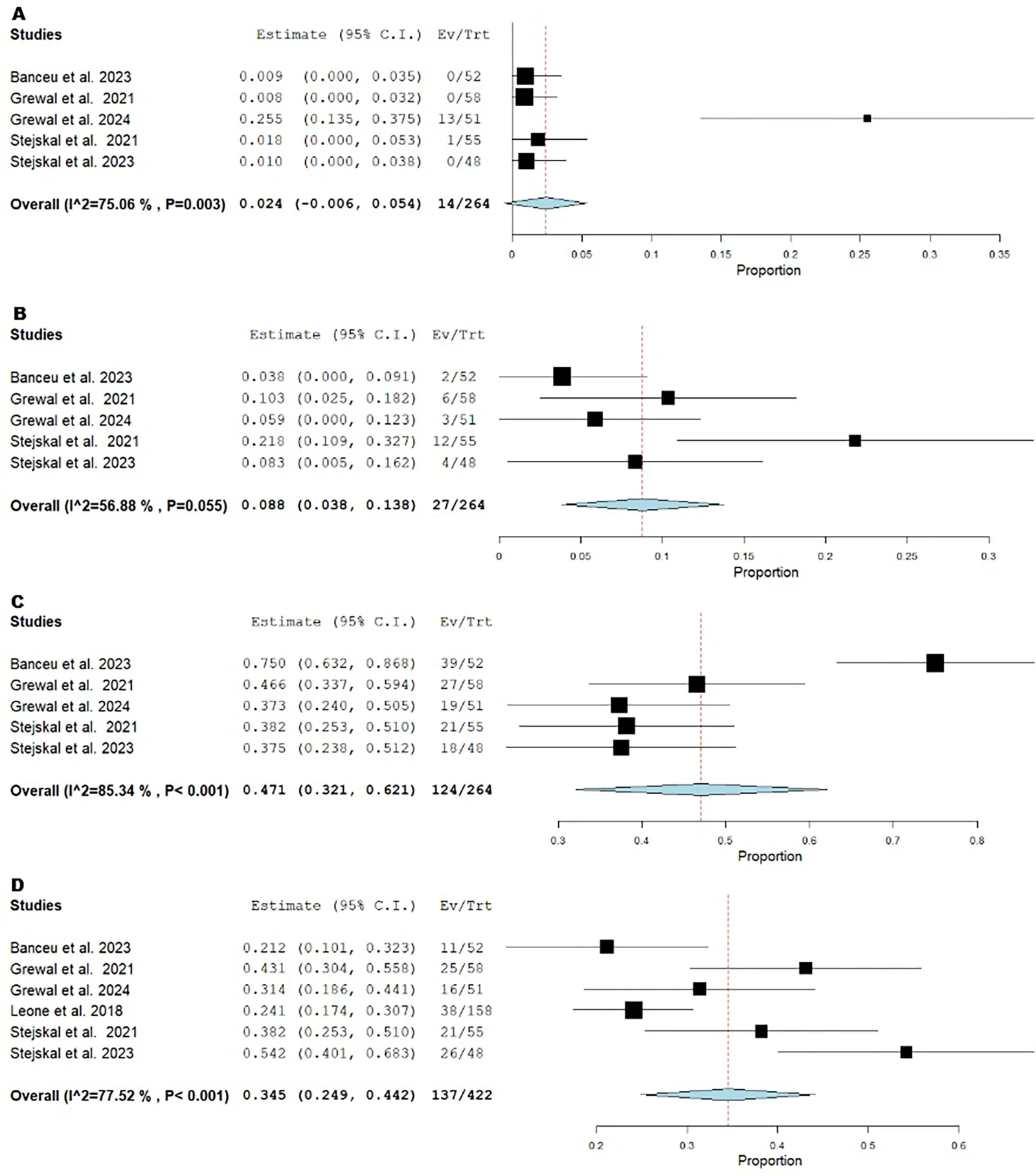
Overall medial degeneration of the whole cohort. A: The pooled prevalence of cases with no medial degeneration among 748 cases combined from eight studies. Out of all cases of combined degeneration, 4.8% (95% CI from 1.7 to 7.8%, *p* < 0.001) were reported without medial degeneration. I^2^ (inconsistency index) was 83.92%. B: The pooled prevalence of cases diagnosed with mild medial degeneration among 748 cases combined from eight studies. Out of all the combined cases, 16.6% (95% CI from 10.2 to 23.0%, *p* < 0.001) were reported with mild medial degeneration. I^2^ was 81.42%. C: The pooled prevalence of cases diagnosed with moderate medial degeneration among 748 cases combined from eight studies. Out of all cases of combined degeneration, 46.8% (95% CI from 41.5 to 52.0%) were reported with moderate medial degeneration. I^2^ was 45.47%. D: The pooled prevalence of cases diagnosed with severe medial degeneration among 748 cases combined from eight studies. Out of all cases combined, 28.9% (95% CI from 21.0 to 36.8%, *p* < 0.001) were reported with severe medial degeneration. I^2^ was 82.01%.

### 3.3 Aneurysm cohort

The most prevalent grade of overall medial degeneration in the aneurysm cohort was moderate, with a prevalence of 48.0% (*n* = 196) in 408 aneurysmatic samples across five studies (95% CI 43.2%–52.8%, with an inconsistency index I^2^ of 0.0%, *p* < 0.001). The second most prevalent grade was severe, with a prevalence of 24.8% (*n* = 80; 95% CI 14.5%–35.1%, I^2^ of 77.75%, *p* < 0.001); 8.2% (*n* = 97) were graded mild. Medial degeneration was absent from only 7.5% (*n* = 35) of the samples (Table 2; Fig. 3). Table 4 summarizes the most prevalent morphological features (Suppl. Fig. 11–20).

**Table 2:** Summary of the severity of medial degeneration among all cohorts.

Cohort	Samples, n	Absent, %	Mild, %	Moderate, %	Severe, %
The whole cohort	748	4.8%	16.6%	46.8%	28.9%
The aneurysm cohort	408	7.5%	18.2%	48.0%	24.8%
The dissection cohort	264	2.4%	8.8%	47.1%	37.2%
The BAV cohort	76	0.8%	43.1%	50.0%	10.6%

**Fig. 3:**
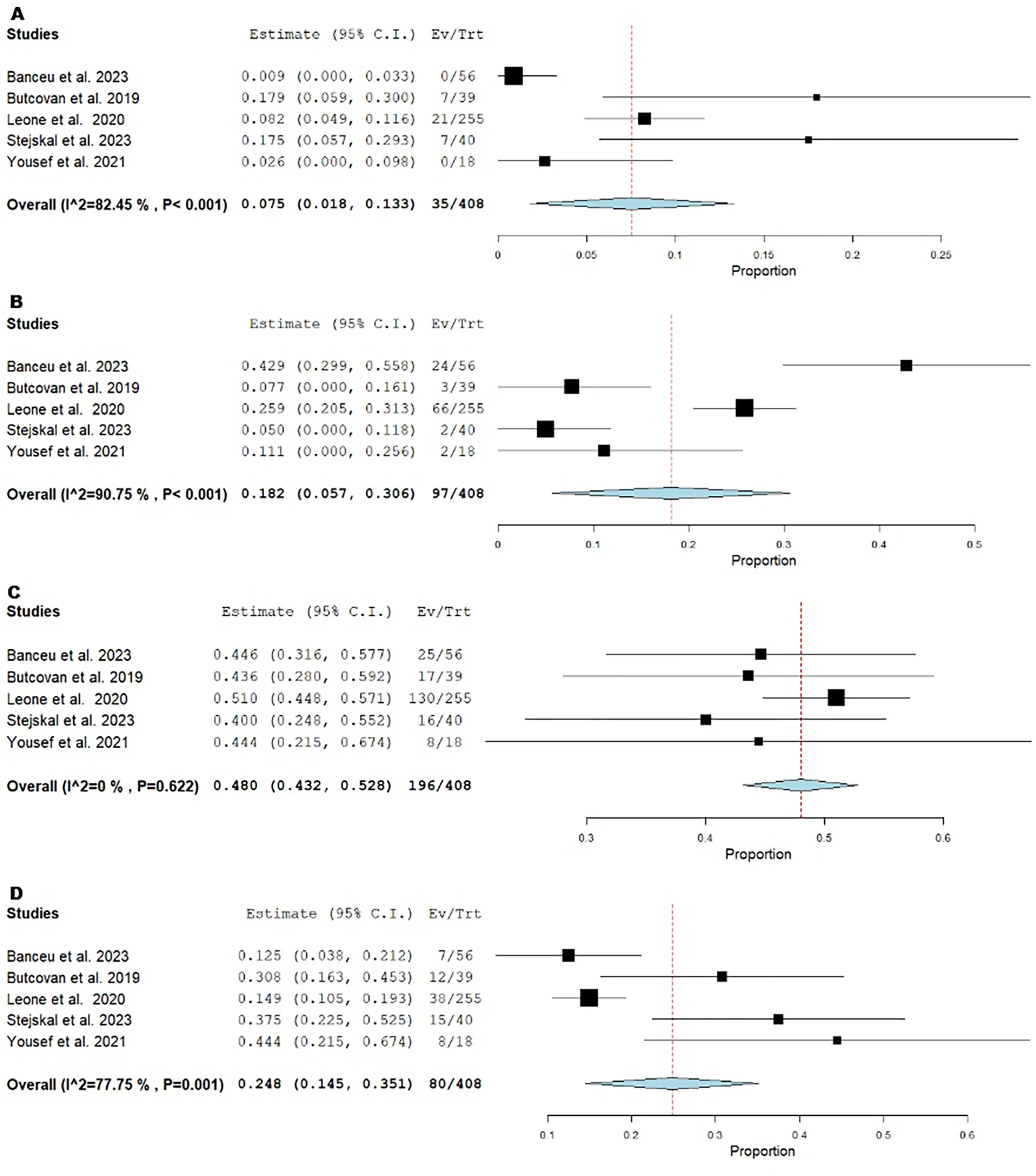
The pooled prevalence of overall medial degeneration in the aneurysm cohort. A: The pooled prevalence of cases with no medial degeneration among 408 cases of aneurysms from five studies. Out of all cases of aneurysm, 7.5% (95% CI, 1.8%–13.3%, *p* < 0.001) were reported without medial degeneration. I^2^ was 82.45%. B: The pooled prevalence of cases diagnosed with mild medial degeneration among 408 cases of aneurysms from five studies. Out of all cases of aneurysms, 18.2% (95% CI from 5.7% to 30.6%, *p* < 0.001) were reported with mild medial degeneration. I^2^ was 90.75%. C: The pooled prevalence of cases diagnosed with moderate medial degeneration among 408 cases of aneurysms from five studies. Out of all cases of aneurysm, 48.0% (95% CI from 43.2 to 52.8%) were reported with moderate medial degeneration. I^2^ was 0.0%. D: The pooled prevalence of cases diagnosed with severe medial degeneration among 408 cases of aneurysms from five studies. Out of all cases of aneurysms, 24.8% (95% CI from 14.5 to 35.1%, *p* < 0.001) were reported with severe medial degeneration. I^2^ was 77.75%.

**Table 3:** Summary of the most prevalent grades of the morphological components of medial degeneration in the whole cohort. (Suppl. Fig. 1–10).

Component	The most prevalent grade	Total number of samples, n	Prevalence %, (n)	95 % Cl, I^2^	P⁎
MEMA-T	Moderate	599	33.8%, (*n* = 219)	17.7%–49.9%, 94.26%	<0.001
MEMA-I	Moderate	599	33.7%, (*n* = 221)	25.4%–42.0%, 75.37%	0.003
EFF/L	Mild	1084	32.5%, (*n* = 257)	18.0%–47.0%, 96.98%	<0.001
EFT	Moderate	598	34.7%, (*n* = 212)	16.1%–53.3%, 96.91%	<0.001
EFD	Absent	345	30.5%, (*n* = 142)	−0.3%–61.2%, 99.67%	<0.001
SMCNL	Moderate	810	32.5%, (*n* = 165)	11.4%–53.6%, 98.04%	<0.001
LMC	Absent	581	57.5%, (*n* = 362)	0.5–84.5%, 98.15%	<0.001
MF-I	Mild	473	49.8%, (*n* = 224)	38.4%–61.1%, 81.84%	–
MF-T	Absent	473	78.0%, (*n* = 347)	63.5%–92.4%, 93.09%	–
Atherosclerosis	Absent	640	52.5%, (*n* = 307)	38.6%–66.5%, 92.61%	–

⁎ Shown when statistically significant.

**Table 4:** Summary of the most prevalent grades of the morphological components of medial degeneration in the aneurysm cohort. (Suppl. Fig. 11–20).

Component	The most prevalent grade	Total number of samples, n	Prevalence %, n	95 % Cl, I^2^	p⁎
MEMA-T	Moderate	369	30.4% (*n* = 141)	14.0–46.9%, 90.68%	<0.001
MEMA-I	Moderate	369	39.2% (*n* = 145)	34.2%–44.1%, 0%	–
EFF/L	Mild	369	44.2% (*n* = 140)	25.3%–63.1%, 0%	<0.001
EFT	Mild	313	48.2% (*n* = 98)	12.9%–83.9%, 96.66%	<0.001
EFD	Absent	58	47.5% (*n* = 37)	−47.5%-135.6%, 99.62%	–
SMCNL	Mild	96	41.3% (*n* = 40)	30.1%–52.4%, 22.45%	–
LMC	Absent	351	55.0% (*n* = 228)	26.4%– 83.7%, 95.61%	–
MF-I	Mild	295	45.9% (*n* = 125)	32.0%–59.8%, 66.58%	–
MF-T	Absent	295	69.4% (*n* = 194)	56.4%–82.5%, 71.45%	–
Atherosclerosis	Absent	334	49.1% (*n* = 156)	25.0%–73.1%, 93.38%	<0.001

⁎ Shown when statistically significant.

**Table 5:** Summary of the most prevalent grades of the morphological components of medial degeneration in the dissection cohort. (Suppl. Fig. 21–30).

Component	The most prevalent grade	Total number of samples, n	Prevalence %, n	95 % Cl, I^2^	p⁎
MEMA-T	Moderate	155	46.6% (*n* = 72)	5.2%–78.1%, 94.97%,	<0.001
MEMA-I	Severe	155	38.6% (*n* = 60)	−4.5%–81.6%, 98.13%,	<0.001
EFF/L	Moderate	264	41.3% (*n* = 110)	19.2%–63.5%, 94.44%,	<0.001
EFT	Moderate	212	45.6% (*n* = 98)	18.5%–72.8%, 95.28%,	<0.001
EFD	Absent	212	48.4% (*n* = 99)	3.5%–93.3%, 99.85%,	<0.001
SMCNL	Mild	263	30.8% (*n* = 82)	17.2%–44.4%, 85.34%,	<0.001
LMC	Absent	155	39.5% (*n* = 62)	2.5%–76.5%, 96.95%,	<0.001
MF-I	Mild	103	43.1% (*n* = 45)	24.0%–62.1%, 75.64%,	0.043
MF-T	Absent	103	81.6% (*n* = 84)	61.9%–102.2%, 85.59%,	0.008
Atherosclerosis	Absent	261	49.6% (*n* = 125)	25.0%–73.1%, 87.21%,	<0.001

⁎ Shown when statistically significant.

**Table 6:** Summary of the most prevalent grades of the morphological components of medial degeneration in the BAV cohort. (Suppl. Fig. 31–39).

Component	The most prevalent grade	Total number of samples, n	Prevalence %, n	95 % Cl, I^2^	p⁎
MEMA-T	Absent	75	65.4% (*n* = 49)	54.6%–76.2%, 0%	–
MEMA-I	Mild	75	46.4% (*n* = 39)	23.0%–69.8%, 50.87%	–
EFF/L	Absent	284	48.9% (*n* = 205)	12.2%–85.7%, 96.53%	<0.001
EFT	Absent	75	74.0% (*n* = 55)	64.2%–83.9%, 0%	–
EFD+	–	–	–	–	–
SMCNL	Mild	284	45.5% (n = 55)	−6.8%–97.7%, 98.52%	<0.001
LMC	Absent	75	96.8% (*n* = 72)	92.8%–100.8%, 0%	–
MF-I	Mild	76	71.1% (*n* = 54)	60.9%–81.2%, 0%	–
MF-T	Absent	75	80.8% (*n* = 69)	44.2%–117.5%, 75.91%	0.042
Atherosclerosis	Absent	76	47.7% (*n* = 26)	8.1%–87.4%, 80.83%	0.022

⁎ Shown when statistically significant.

+ Not enough data for analysis.

### 3.4 Dissection cohort

The most prevalent grade of overall medial degeneration in the dissection cohort was moderate, with a prevalence of 47.1% (*n* = 124) in 264 dissection samples across five studies (95% CI 5.7%–30.6%, with an inconsistency index of 85.34%, *p* < 0.001). The second most prevalent grade was severe, with 37.2% (*n* = 99) samples (95% CI 26.2%–48.2%, I^2^ of 73.45%, *p* < 0.001). Only 8.8% were graded as mild (*n* = 27), and in 2.4% (*n* = 14) of the samples, medial degeneration was absent (Table 2; Fig. 4). Table 5 summarizes the most prevalent morphological features (Suppl. Fig. 21–30).

**Fig. 4:**
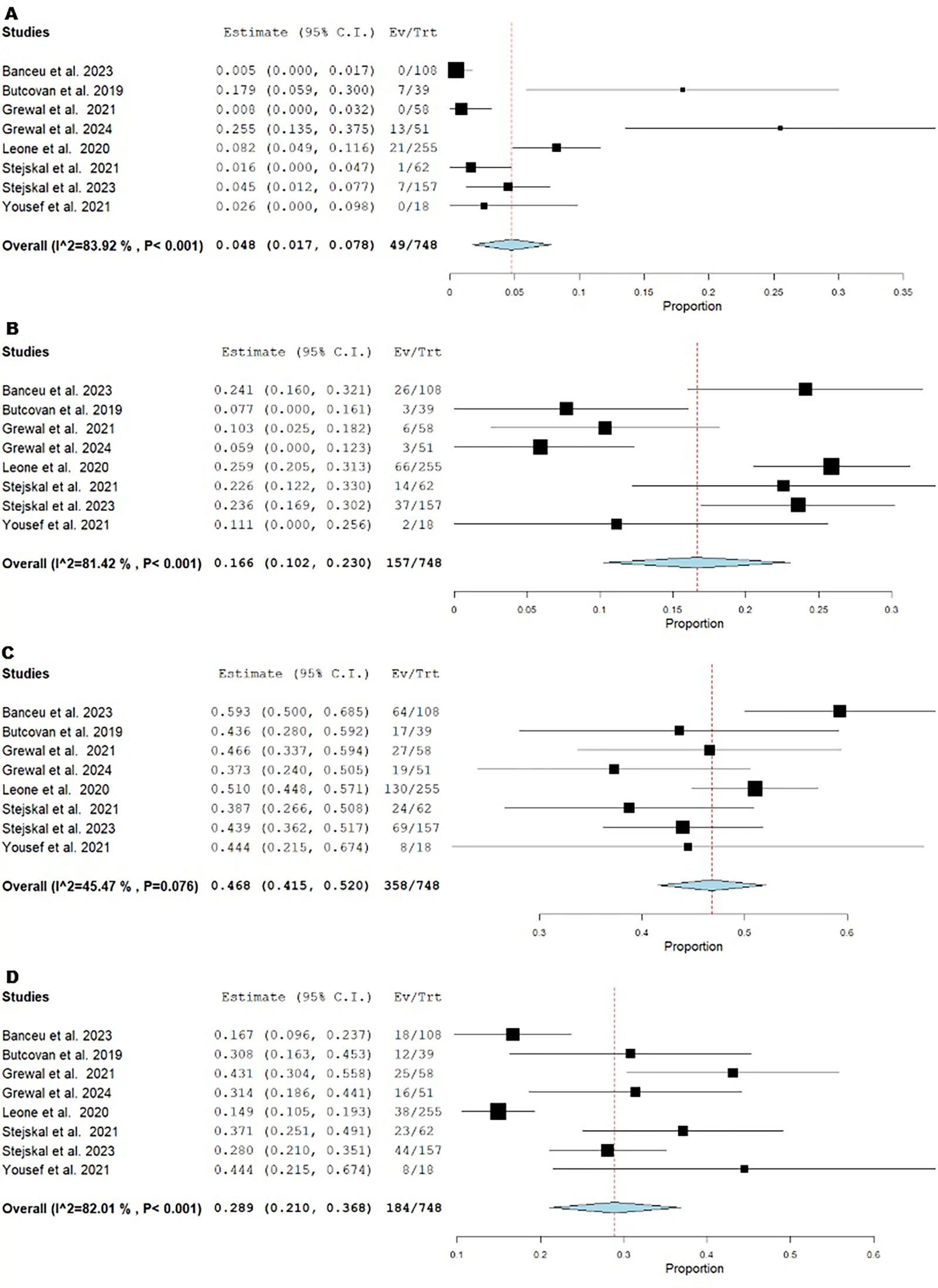
The pooled prevalence of medial degeneration in the dissection cohort. A: The pooled prevalence of cases with no medial degeneration among 264 cases of dissection from five studies. Of all cases of dissection, 2.4% (95% CI from −0.6 to 5.4%, *p* = 0.003) lacked medial degeneration. I^2^ was 75.06%. B: The pooled prevalence of cases diagnosed with mild medial degeneration among 264 cases of dissection from five studies. Out of all cases of dissection, 8.8% (95% CI from 3.8% to 13.8%) were reported with mild medial degeneration. I^2^ was 56.88%. C: The pooled prevalence of cases diagnosed with moderate medial degeneration among 264 cases of dissection from five studies. Out of all cases of dissection, 47.1% (95% CI from 32.1% to 62.1%, *p* < 0.001) were reported with moderate medial degeneration. I^2^ was 85.34%. D: The pooled prevalence of cases diagnosed with severe medial degeneration among 264 cases of dissection from five studies. Out of all cases of dissection, 37.2% (95% CI from 26.2% to 48.2%, *p* < 0.001) were reported with severe medial degeneration. I^2^ was 73.54%.

### 3.5 Bicuspid aortic valve cohort

The most prevalent grade of overall medial degeneration in the BAV cohort was moderate, with a prevalence of 50% (*n* = 38) in 78 bicuspid aortic valve samples across two studies (95% CI 38.8%–61.2% with an inconsistency index of 0%). The second most prevalent grade was mild in 43.1% (*n* = 33) of the samples (95% CI 32.1%–54.2%, I^2^ of 0%). Only 10.6% (*n* = 5) were graded as severe, and in 0.8% (*n* = 0) of samples, medial degeneration was absent (Table 2; Fig. 5). Table 6 summarizes the most prevalent morphological features (Suppl. Fig. 31–39).

**Fig. 5:**
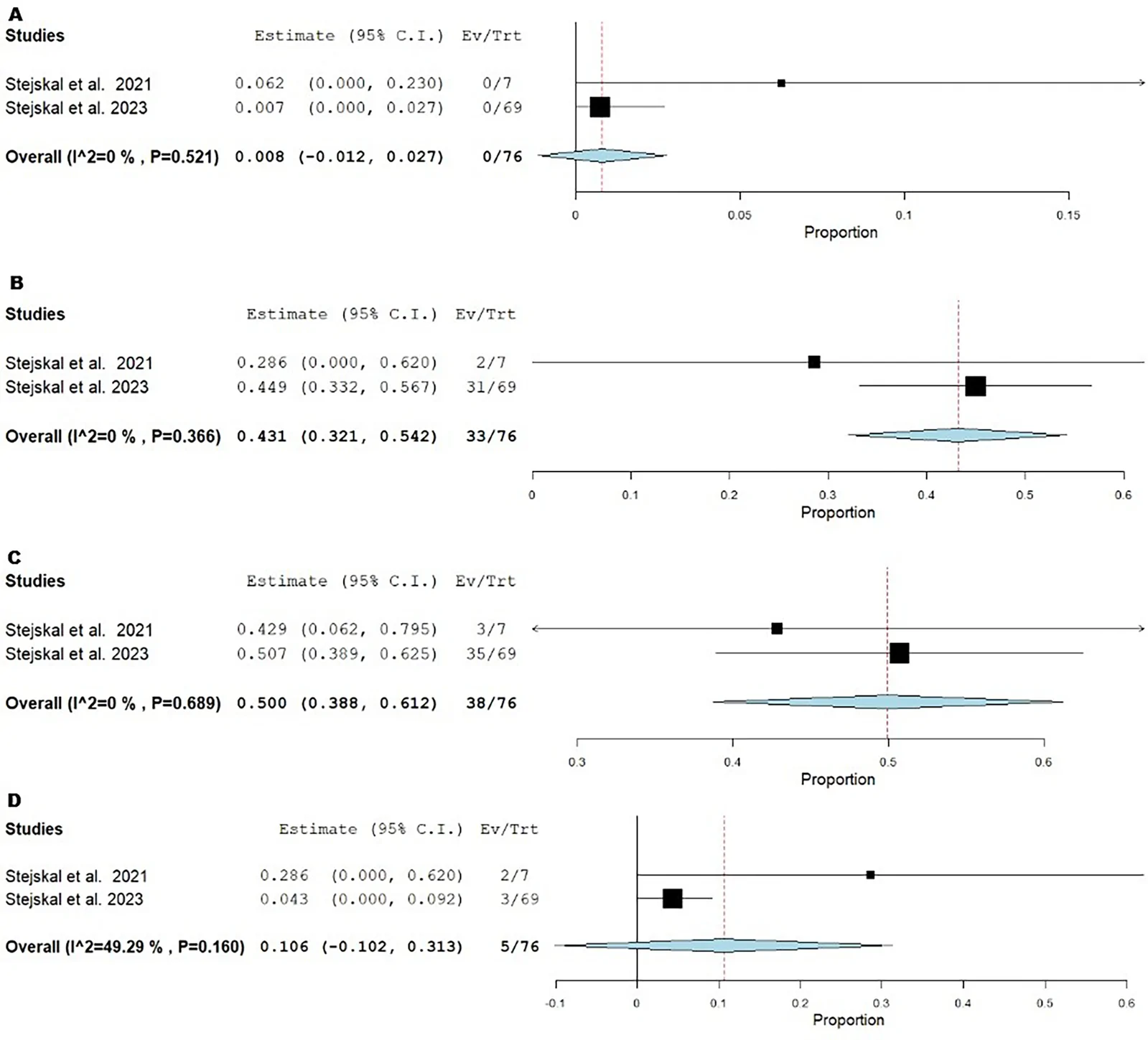
The pooled prevalence of medial degeneration in the BAV cohort. A: The pooled prevalence of cases with no medial degeneration among 76 cases of BAV from two studies. Of all cases of BAV, 0.8% (95% CI from −1.2% to 2.7%, *p* = 0.008) were reported without medial degeneration. I^2^ was 0%. B: The pooled prevalence of cases diagnosed with mild medial degeneration among 76 cases of BAV from two studies. Out of all cases of BAV, 43.1% (95% CI from 32.1% to 54.2%) were reported with mild medial degeneration. I^2^ was 0%. C: The pooled prevalence of cases with moderate medial degeneration among 76 cases of BAV from two studies. Out of all cases of BAV, 50.0% (95% CI from 38.8% to 61.2%) were reported with moderate medial degeneration. I^2^ was 0%. D: The pooled prevalence of cases diagnosed with severe medial degeneration among 76 cases of BAV from two studies. Of all cases of BAV, 10.6% (95% CI from −10.2% to 31.3%) were reported with severe medial degeneration. I^2^ (inconsistency index) was 0%.

### 3.6 Results divided based on consensus morphological categories

#### 3.6.1 Medial degeneration

Meta-analyses of thoracic aortic surgical samples classified according to the SCVP and AECVP consensus statements showed overall moderate to severe medial degeneration with more severe degeneration than mild and absent degeneration combined (Table 2). Only 4.8% of the samples in the whole cohort revealed no medial degeneration. Almost half of the samples had moderate medial degeneration with a prevalence of 46.8%, and the rest were distributed with 16.6% as mild and 28.9% as severe degeneration. In our meta-analysis, the dissection cohort depicted slightly more severe overall degeneration (34.5%) than the 24.8% in the aneurysm cohort. The BAV cohort displayed the mildest overall degeneration (Table 2, Fig. 6A).

**Fig. 6A:**
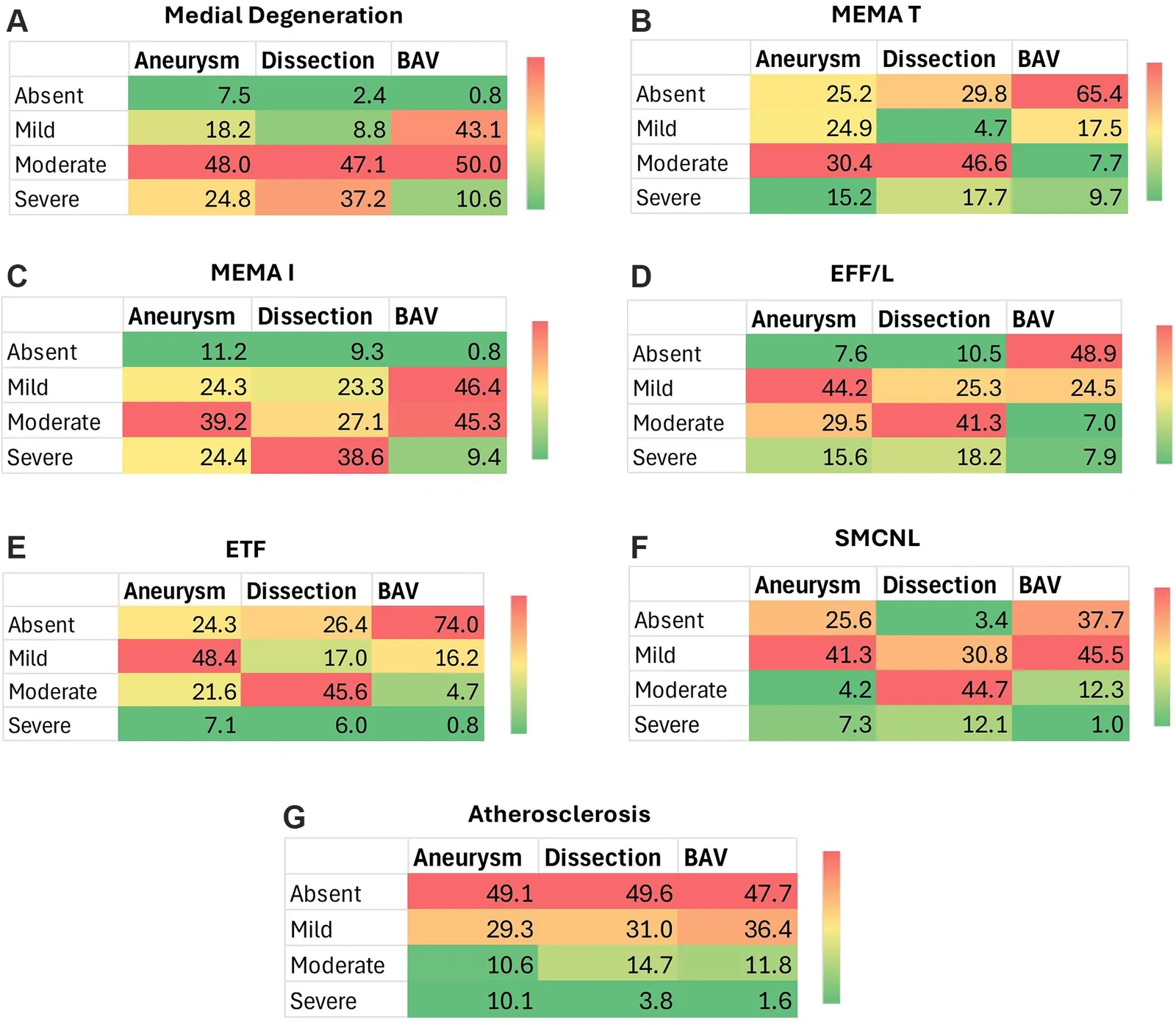
Heat maps of major histopathological features accros aneurysmtaic, dissection, and BAV cohorts. A. heat-map of the pooled prevalence (%) of samples in each severity grade of medial degeneration among cohorts. BAV, bicuspid aortic valve. B. A heat-map of the pooled prevalence (%) of samples in each severity grade of translamellar mucoid extracellular matrix accumulation among cohorts. BAV, bicuspid aortic valve, MEMA T translamelar medial extracellular matrix accumulation. C. A heat-map of the pooled prevalence (%) of samples in each severity grade of intralamellar mucoid extracellular matrix accumulation among cohorts. BAV, bicuspid aortic valve, MEMA I Intralamelar medial extracellular matrix accumulation. D. A heat-map of the pooled prevalence (%) of samples in each severity grade of elastic fiber fragmentation and/or loss among cohorts. BAV, bicuspid aortic valve, EFF/L elastic fiber fragmentation/loss. E. A heat-map of the pooled prevalence (%) of samples in each severity grade of elastic fiber thinning among cohorts. BAV, bicuspid aortic valve, EFT elastic fiber thinning. F. A heat-map of the pooled prevalence (%) of samples in each severity grade of smooth muscle cell nuclei loss among cohorts. BAV, bicuspid aortic valve, SMCNL smooth muscle cell nuclear loss. G. A heat-map of the pooled prevalence (%) of samples in each severity grade of atherosclerosis among cohorts. BAV, bicuspid aortic valve.

#### 3.6.2 Translamellar mucoid extracellular matrix accumulation

In the whole cohort, MEMA-T was present in 66.9% of the samples. MEMA-T was found in 74.8% of the samples in the aneurysm cohort and in 70.2% of the dissection cohort, but only in 44.6% of the BAV cohort. Most samples (46.6%) in the dissection cohort were graded moderate, compared to 30.4% in the aneurysm cohort. Only 4.7% of samples in the dissection cohort were graded mild, compared to 24.9% in the aneurysm cohort. The BAV cohort showed less severe changes in MEMA-T than the other cohorts, with 17.5% of samples graded mild and 7.7% moderate. The prevalences between the aneurysm and dissection cohorts were similar. However, dissection tended to have more severe changes in the MEMA-T. In the BAV cohort, MEMA-T was rarer and less severe than in other cohorts (Fig. 6B; Suppl. Fig. 1, 11, 21, 31).

#### 3.6.3 Intralamellar mucoid extracellular matrix accumulation

Overall, MEMA-I was more prevalent and more severe than MEMA-T. MEMA-I was present in 88.8% of samples in the aneurysm cohort and in 90.7% in the dissection cohort. MEMA-I also mirrors MEMA-T in severity differences between cohorts, as the dissection cohort showed more severe changes in MEMA-I than the aneurysm cohort. MEMA-I was graded severe in 38.6% of the samples in the dissection cohort and 24.4% in the aneurysm cohort. BAV cohort showed less severe grades of MEMA-I. However, it had a higher overall prevalence. MEMA-I had a prevalence of 100% in the BAV cohort, with mild- and moderate-grade MEMA-I making up 91.7% of the samples. However, the BAV cohort included only a few samples (*n* = 75) (Fig. 6C; Suppl. Fig. 2, 12, 22, 32).

#### 3.6.4 Elastic fiber fragmentation and/or loss

EFF/L was the largest single component in our analysis, with a total of 1084 samples included. EFF/L was found in 80.6% of the samples, and the most prevalent grade was mild, with a prevalence of 32.5%. EFF/L was absent from 48.9% of cases in the BAV cohort, compared to 7.6% in the aneurysm cohort and 10.5% in the dissection cohort. The dissection cohort had a prevalence of 89.5% for EFF/L. We also found slight differences in severity among cohorts. EFF/L was mild to moderate in the aneurysm cohort, moderate in the dissection cohort, and mild to absent in the BAV cohort **(**Fig. 6D; Suppl. Fig. 3, 13, 23, 33).

#### 3.6.5 Elastic fiber thinning

The most prevalent grade of EFT was moderate, with a prevalence of 34.7%. However, the second most common grade was mild, with a prevalence of 30.8%. The BAV cohort revealed the least severe changes, with EFT being absent from 74.0% of the samples and 16.2% being mild. The most severe changes were found in the dissection cohort, with 45.6% of samples having moderate changes and 17.0% having mild changes. EFT was milder in the aneurysm cohort than in the dissection cohort, with 48.4% of samples depicting mild changes and 21.6% displaying moderate changes (Fig. 6E; Suppl. Fig. 4, 14, 24, 34).

#### 3.6.6 Elastic fiber disorganization

EFD was present in 60.5% of the samples in the whole cohort and was most often multifocal, with a prevalence of 23.2%. The overall prevalence between cohorts was similar: 52.5% in the aneurysm cohort and 51.6% in the dissection cohort. There was insufficient data to analyze EFD in the BAV cohort. EFD was focal in 29.0% of the samples in the aneurysm cohort and in 9.2% of dissections. A more severe change, multifocal, was almost equally present in both cohorts, with 21.9% of the samples in the aneurysm cohort and 19.5% in dissections. Only 1.5% of the samples in the aneurysm cohort had extensive EFD, compared to 10.6% in the dissection. The extent of EFD continues earlier trends, as the aneurysm cohort shows a less severe extent than dissections (Suppl. Fig. 5, 15, 25).

#### 3.6.7 Smooth muscle cell nuclei loss

In the whole cohort, SMCNL was found in 77.0% of samples and was most often moderate to mild in severity, with a prevalence of 32.5% for the moderate grade and 29.3% for the mild grade. In our analysis, there was a difference in prevalence and severity between cohorts. The aneurysm cohort had a prevalence of 77.0% for SMCNL, which was closer to the BAV cohort's prevalence of 62.3% than to the dissection's 96.6%. SMCNL in the current meta-analysis seems associated with dissection more than the other two conditions, as 44.7% of the dissection cohort was graded moderate. SMCNL was mostly mild in both the aneurysm cohort and the BAV cohort, with prevalences of 41.3% and 45.5%. The aneurysm cohort had a small sample size of 96 in this analysis, which may explain the difference between SMCNL and other sub-components in the aneurysm cohort (Fig. 6F; Suppl. Fig. 6, 16, 26, 35).

#### 3.6.8 Smooth muscle cell disorganization

The articles chosen for the current meta-analysis did not provide sufficient data on SMCD to investigate this sub-component of medial degeneration.

#### 3.6.9 Laminar medial collapse

Overall, LMC was a rarer component. It was present in 46.5% of all samples in the whole cohort and was almost equally split between the mild and the moderate grades, with 17.6% of samples graded mild and 17.3% moderate. In the BAV cohort, LMC was almost absent, with a prevalence of 3.2%. The aneurysm cohort had a prevalence of 45.0% and dissections of 60.5%. LMC was graded moderate in 20.7% and severe in 1.7% of the samples in the aneurysm cohort. The dissection cohort demonstrates more severe changes in LMC than aneurysms, with 19.7% of samples graded moderate and 12.6% severe (Suppl. Fig. 7, 17, 27, 36).

#### 3.6.10 Intralamellar medial fibrosis

MF-I was present in 89.4% of all samples, with 49.8% graded mild and 33.1% graded moderate. The BAV cohort showed more prevalent MF-I than the other cohorts, with a prevalence of 99.2% compared to 84.0% in the aneurysm cohort and 91.8% in the dissection cohort. However, the BAV cohort still showed less severe changes in MF-I, with 72.0% of samples graded mild compared to 45.9% of the samples in the aneurysm cohort and 43.1% in the dissection cohort (Suppl. Fig. 8, 18, 28, 37).

#### 3.6.11 Translamellar medial fibrosis

MF-T was prevalent in 22.0% of the samples in the whole cohort. The severity was similar among the cohorts. However, MF-T was more prevalent in the aneurysm cohort than in the dissection cohort or the BAV cohort. MF-T was present in 19.2% of the samples in the BAV cohort, 18.4% in the dissection cohort, and 30.6% in the aneurysm cohort (Suppl. Fig. 9, 19, 29, 38).

#### 3.6.12 Aortitis

We could not analyze the aortitis due to a lack of sufficient data in the analysed articles.

#### 3.6.13 Atherosclerosis

Atherosclerosis was present in 52.5% of the 670 samples and was most commonly mild in severity (30.0%) in the whole cohort. The prevalence of atherosclerosis was almost identical in all three cohorts, with a prevalence of 49.9% in the aneurysm cohort, 49.6% in the dissection cohort, and 52.3% in the BAV cohort. The severity of atherosclerosis was also similar among all cohorts (Fig. 6G; Suppl. Fig. 10, 20, 30, 39).

## 4 Discussion

This meta-analysis of surgically removed thoracic aortic samples showed that there was significant overlap in the histopathology of dissected, aneurysmatic, and BAV samples. Only slight differences can be found. In the whole cohort, MEMA was present with moderate severity. MEMA-I was more prevalent than MEMA-T. MF-I was one of the most common components, with an overall prevalence similar to MEMA-I. MF-T and MF-I followed a similar pattern as MEMA-T and I, where the translamellar type is less prevalent and the intralamellar type is one of the more common subcomponents. Changes in elastic fibers were moderate to mild in severity, and MF was mild in severity. SMCNL was generally moderate.

The dissection cohort showed more severe medial degeneration than the aneurysm cohort or the BAV cohort. In the current analysis, the prevalence and severity of SMCNL in the dissection cohort was considerably higher than in the other two analysed cohorts. Not as significant, but still greater distinctions were observed in EFF/L, EFT, and EFD. Smaller or insignificant differences were seen in MEMA-T, MEMA-I, LMC, MF-I, and MF-T. The aneurysm cohort showed less severe findings than the dissection cohort. The BAV cohort showed consistently less severe features of medial degeneration, especially in EFF/L and EFT, which were much less common findings in the BAV aortas than in the dissected and aneurysmatic aortas. Atherosclerosis was similar among all cohorts.

Huhta et al. [9] analysed 217 surgical thoracic ascending aorta specimens, including dilated, aneurysmatic, and dissected samples. All cases had medial degeneration. They observed a slightly less severe distribution in severity, as 20.5% of their samples were graded mild, 54.0% moderate, and 25.6% severe. Overall, their results for medial degeneration corroborated the combined whole cohort in the current meta-analysis. For the features of medial degeneration, they found a lower prevalence for SMCNL (67.6%), EFT (63.6%), and LMC (37.3%) and a considerably lower prevalence for MF (13.4%). Additionally, they found a higher prevalence of EFF/L (95.4%) and MEMA (100%). Their study's population was similar in age and sex distribution to ours, and they found that dissected aortas had primarily moderate to severe medial degeneration than aneurysmatic aortas, which also included a proportion of mild medial degeneration. In the current analysis, dissection was also associated with more severe degeneration, in alignment with Huhta et al. [9].

Amemiya et al. [7] analysed 191 BAV, 137 dissected, and 168 aneurysmatic aortic samples. They used a scoring system to report their findings, which makes direct comparison difficult, though it is possible to identify patterns in results. First, they found a similar pattern in which BAV patients had less severe medial degeneration than the aneurysm and dissection cohorts. Additionally, they found that medial degeneration significantly increased in the dissected aortas compared to the aneurysmatic aortas. These results can also be witnessed in our meta-analysis. Their study's population was similar to ours in terms of age and sex distribution.

Tucker et al. [6] analysed 314 samples of aneurysmatic aortas and 86 aortas from patients with BAV. Part of the study's population was evaluated before the publication of the consensus documents. Nevertheless, the authors recategorized the histopathological findings according to the new nomenclature. They did not find differences in EFF/L between the aneurysmatic and BAV samples, unlike in the current analysis, where the BAV cohort had less prevalent changes in the elastic fibers. Additionally, they found higher frequencies of MF and SMCNL in the BAV population. In the current analysis, the SMCNL was almost the same in the aneurysm cohort and the BAV cohort. MF-I was more prevalent in the BAV cohort. However, when MF-I was present, it was more severe in the aneurysm cohort.

Chen et al. [26] analysed 141 samples of dissected aortas, all of which had medial degeneration, which was only slightly higher than in the current analysis. Chen et al. used a scoring system from 0 to 3 to grade the attributes of medial degeneration. They found a severity score of 2.4 for medial degeneration, meaning moderate to severe changes, which corresponds with the current meta-analysis. In the subcomponents of medial degeneration, they found a higher prevalence of 100% for EFF/L and 79.4% for EFT than the current analysis and a slightly lower prevalence of 61.0% for EFT, 86.5% for SMCNL, 55.3% for LMC, and significantly lower 13.5% for MF. Apart from MF, the prevalence nears the prevalence found in this analysis. Characteristics of their cohort also reflect those of the current analysis, with a median age of 64 and two-thirds of samples from male patients. They concluded that changes in elastic fiber composition may expose the aorta to dissection, although we observed that the SMCNL could be more tightly linked to dissections.

The BAV cohort showed less severe findings in all the features of medial degeneration apart from MF-I, where it had the highest prevalence compared to the other cohorts. However, the differences in MF-I between cohorts were small. Similar results were found in a recent article by Buja et al. [21], who found significantly less severe EFF/L and milder medial degeneration in samples from patients with BAV than tricuspid aortic valve (TAV) samples. In other studies, Mennander et al. [8] found that samples from patients with TAV had significantly more prominent medial degeneration than samples from patients with BAV, and dilatation was not associated with increased medial degeneration in BAV samples. Amemiya et al. [7] found that a larger aortic diameter correlated with more severe medial degeneration in samples from patients with or without BAV.

BAV is considered a risk factor for aortic dilatation, so these patients often undergo surgical treatment earlier, with smaller aortic diameters than patients with TAV. This may explain the differences in generally milder histopathological findings between samples from patients with BAV and TAV. Nevertheless, the correlation between aortic diameter and medial degeneration is contested, which is why a multivariable assessment of the patient's need for surgery is recommended [2], [22]. Nevertheless, TAV seems to be associated with more severe medial degeneration compared to BAV in this meta-analysis and in the previous literature [7], [8], [21], [23].

Noninfectious aortitis is an inflammatory process involving the aortic wall that is not explained by atherosclerosis or infection. Some specific entities linked to aortitis are giant cell arteritis, Takayasu arteritis, IgG4-related disease, and sarcoidosis. Several articles have shown an association between aortitis and medial degeneration in aortic samples. Tucker et al. [6] found a prevalence of 7.3%, Butcovan et al. [12] 12.8%, Leone et al. [17] 13.7%, and Stejskal et al. [19] 20% of aortitis in the aneurysmatic aortas. Amemiya et al. [24] found that aortitis causes significant damage to the aorta and is linked to more severe medial degeneration. Leone et al. [17] also concluded that inflammation contributes to the development of thoracic aorta disease. Aortitis was often an unexpected diagnosis before surgery, and inflammation significantly contributed to thoracic aortic disease, either as an independent factor or together with medial degeneration and atherosclerosis [6], [17], [24]. Older age has also been linked to aortitis in the literature [6], [24]. Recently, Buja et al. [21] concluded that even though they found a lower prevalence of aortitis, the inflammatory process contributed to the development of thoracic aortic aneurysms. We could not analyze aortitis in the current meta-analysis. Nonetheless, there seems to be a growing need to recognize the silent but damaging process of subclinical inflammation in the preoperative diagnosis.

The previous understanding was that atherosclerosis increases distally from the ascending aorta. In a recent review by Buja et al. [22], the authors concluded that simultaneous medial degeneration and atherosclerosis in the ascending aorta is rare. In their review, Waldron et al. [25] provided evidence that a thoracic aorta aneurysm protects from the atherosclerotic process. In the current analysis, there were similar results, with mostly mild atherosclerosis occurring in approximately one in three samples. Huhta et al. [9] found a higher prevalence of 75.8% for atherosclerosis in a series of 217 ascending aortic samples; furthermore, severe atherosclerosis was connected to more severe medial degeneration. This higher prevalence can be explained by the unique population in their series. Chen et al. [9] found a prevalence of 76.7% in 141 samples from patients undergoing dissection surgery, which is higher than in the present meta-analysis (49.6%). As all cohorts in the current analysis showed similar atherosclerosis, it may be speculated that atherosclerosis is formed independently of other components of medial degeneration.

The classification scheme provided by the consensus documents allows for an extensive evaluation of the medial degeneration of aortic samples. Based on the published articles unified nomenclature and definitions have been found helpful for systematic evaluation of these samples. As the relationship between individual lesions of medial degeneration and the clinical manifestations is unclear, reporting the histopathological changes in the samples comprehensively is important, so that further multi-institutional research and meta-analyses can establish these relationships. Unfortunately, some lesions such as MF and SMCD are often left out from the results in published articles which can leave crucial information out of meta-analysis. The consensus documents seem to capture the most relevant morphological features so far reported in the medial layer of the thoracic aorta. Analysed and discussed articles did not describe any additional features not covered by the consensus documents nor did the studies make note of potential lacking features in the consensus documents [11], [12], [13], [14], [15], [16], [17], [18], [19], [20].

## 5 Limitations

This study has limitations that weaken the conclusions that can be drawn out from the data. The number of eligible studies for analysis is small. This is partly because the classification scheme is relatively new and the research surrounding this subject is still ongoing but more due to the incompatible styles of reporting results in the published literature. There were two popular formats. A frequency-based system that shows how many samples fall into a specific severity grade. This is the format used in this analysis and therefore in the eligible studies. Another style of reporting was a graded system which gives a value for each severity grade, usually from 0 to 4, where 0 is absent and 4 severe and then calculates a score for each of the subcomponents of medial degeneration, i.e. “*The severity of EFF/L in the aneurysm cohort was 2.4”*. This way of presenting the results is intuitive and allows easy comparison between cohorts but is incompatible for analysis with the former way and therefore studies using this method were excluded. At least six studies were excluded for this reason. A unified method of presenting results could strengthen future research and analyses.

Additionally, some articles do not report on all components of medial degeneration, which results in varied sample sizes in the analyses. These component analyses included more than 500 specimens and comprised all cohorts: MD, MEMA-T and MEMA-I, EFF/L, EFT, SMCNL, atherosclerosis, and LMC. Components with fewer data were EFD, SMCD, MF-T, MF-I, and aortitis. No explanation was provided for why some components were excluded from the results of the articles. Additionally, studies have often reported MEMA and MF without distinguishing between translamellar and intralamellar types. Hence, an analysis between these two types of reporting was not possible. Our analysis did not compare dissections with pre-existing aneurysms to dissections alone. Therefore, the role of medial degeneration in pure dissections is unclear.

Several analyses demonstrated substantial statistical heterogeneity despite the use of random-effects models. This heterogeneity likely reflects both methodological and biological variability among the included studies. Although all studies used the SCVP/AECVP consensus nomenclature, differences existed in the practical implementation of grading systems, histopathological evaluation, and reporting style. In addition, inter-observer variability, differences in tissue sampling and staining methods, as well as clinical heterogeneity of the analysed cohorts may have contributed to the observed variability. Finally, several subgroup analyses included only a limited number of studies and relatively small sample sizes, particularly in the BAV cohort, which may have further increased statistical heterogeneity and limited the generalizability of some pooled estimates.

Only surgically treated samples with histopathological analysis were included. Due to missing and/or inconsistent data on other aortic valve malformations and connective tissue disorders, such as Marfan, Turner, Ehler-Danlos, and Loyes–Dietz syndromes, they were not included and separately investigated.

Section 3.1 demonstrated that we could not remove all samples from patients with BAV from our aneurysm and dissection cohorts, which resulted in confounding among the aneurysm, dissection, and BAV cohorts. Additionally, the BAV cohort is made of samples removed for different indications and includes aneurysmatic, dissected, and dilated aortic samples.

There is a search methodological limitation. The PubMed “cited by” search is not a comprehensive method due reasons unknown to the authors and it does not find all the articles citing the consensus documents in the MEDLINE database. Therefore, we added six articles that do exist in the database but were not found in the primary search. It may introduce a selection bias in the analysis. As this analysis includes studies from the same institutions, there is a possibility of overlapping in cohorts. Furthermore, some articles did not disclose the sample collection period and there is overlap in the collection periods between Stejskal et al. 2021 [18] and Stejskal et al. 2023 [19].

As the search ended in March 2024, newer articles are not included in the analysis [9], [21]. Furthermore, several studies were not analysed [6], [7], [9], [25], [26] due to different data formats and currently more up-to-date research may be available.

## 6 Conclusion

Almost every surgically removed aortic sample exhibited medial degeneration, which represents the pathological process in thoracic aortic disease. We could not strongly link any specific component of medial degeneration to any surgical cohort which indicates a shared pathophysiological process common to these surgical conditions. We confirmed earlier findings that samples from patients with BAV have less severe medial degeneration. When compared to the aneurysm cohort, the dissection cohort had more severe changes across several components and, therefore, overall medial degeneration. More research is needed to confirm these findings.

The consensus documents emphasized the importance of the routine and systematic analysis of resected samples [5]. This supports further research into the histopathological characteristics of surgically removed aortic samples and the underlying conditions that compromise the integrity of the aortic wall. A uniform and consistent way of reporting these insights is encouraged to increase the potential of future meta-analyses. Consensus documents assist the process of systematic review by providing clear definitions and a useful reporting tool that helps organize and collect information.

This meta-analysis is just the first step to receive a more complex view of the morphological features connected with various thoracic aorta pathologies. We were able to find small differences between cohorts and confirm some earlier findings, but these insights are not yet enough to draw solid conclusions or clinical guidelines on managing thoracic aortopathies. Further multi-institutional research with larger series and their meta-analyses are needed for more concrete results. In the future, cardiothoracic surgeons may be able to plan the extent and follow up of surgical interventions better with the help of histopathological analysis of the thoracic aortic tissue.

## CRediT authorship contribution statement

**Eemeli Jämsen:** Writing – original draft, Methodology, Formal analysis, Data curation, Conceptualization. **David Kalfeřt:** Writing – review & editing, Writing – original draft, Visualization, Validation, Software, Resources, Methodology, Investigation, Formal analysis, Data curation. **Tero Halmesmäki:** Writing – review & editing, Formal analysis, Data curation. **Ari Mennander:** Writing – review & editing, Supervision, Formal analysis. **Timo Paavonen:** Writing – review & editing, Conceptualization. **Ivana Kholová:** Writing – review & editing, Writing – original draft, Validation, Supervision, Resources, Project administration, Methodology, Investigation, Funding acquisition, Conceptualization.

## Declaration of ethical statement

The authors declare that the study is a compilation of published data and as such do not require ethical committee approval.

## Funding

VTR Competitive Research Grant from Pirkanmaa Wellbeing Services County, the Aarne Koskelo Foundation, the Foundation of the Finnish Anti-Tuberculosis Association, the Tampere Tuberculosis Foundation, and the Cooperatio Program, research area SURG.

## Declaration of competing interest

The authors declare that they have no conflicts of interest.

## Data Availability

All data are included in the article and supplementary material.
