# Supplementary material for: Meta-analysis of the histopathological features of surgically removed aortas classified according to the SCVP and AECVP consensus documents
Source: Am Heart J Plus. 2026 Sep 25;71:100904. doi: 10.1016/j.ahjo.2026.100904 (PMC13654543; doi:10.1016/j.ahjo.2026.100904)
Supplement: Supplementary file 1 [file mmc1.docx]

Supplementary figures

The whole cohort

The whole cohort - Translamellar mucoid extracellular matrix accumulation

Suppl. Fig. 1. The whole cohort - Translamellar mucoid extracellular matrix accumulation.

A: The pooled prevalence of cases diagnosed MEMA T as absent among 599 cases of combined from 5 studies. Out of all cases of combined, 33.1% (95% CI from 14.4 to 51.7%) were reported MEMA T as absent. I^2^ (inconsistency index) was 97.58%. B: The pooled prevalence of cases diagnosed MEMA T as mild among 599 cases of combined from 5 studies. Out of all cases of combined, 18.8% (95% CI from 3.6 to 34.0%) were reported MEMA T as mild. I^2^ (inconsistency index) was 97.15%. C: The pooled prevalence of cases diagnosed MEMA T as moderate among 599 cases of combined from 5 studies. Out of all cases of combined, 33.8% (95% CI from 17.7 to 49.9%) were reported MEMA T as moderate. I^2^ (inconsistency index) was 94.26%. D: The pooled prevalence of cases diagnosed MEMA T as severe among 599 cases of combined from 5 studies. Out of all cases of combined, 14.7% (95% CI from 11.8 to 17.5%) were reported MEMA T as severe. I^2^ (inconsistency index) was 1.34%.

The whole cohort - Intralamellar mucoid extracellular matrix accumulation

Suppl. Fig. 2. The whole cohort - Intralamellar mucoid extracellular matrix accumulation

A: The pooled prevalence of cases diagnosed MEMA I as absent among 599 cases of combined from 5 studies. Out of all cases of combined, 10.1% (95% CI from 1.5 to 18.9%) were reported MEMA I as absent. I^2^ (inconsistency index) was 95.09%. B: The pooled prevalence of cases diagnosed MEMA I as mild among 599 cases of combined from 5 studies. Out of all cases of combined, 31.8% (95% CI from 1.7 to 47.9%) were reported MEMA I as mild. I^2^ (inconsistency index) was 95.56%. C: The pooled prevalence of cases diagnosed MEMA I as moderate among 599 cases of combined from 5 studies. Out of all cases of combined, 33.7% (95% CI from 25.4 to 42.0%) were reported MEMA I as moderate. I^2^ (inconsistency index) was 75.37%. D: The pooled prevalence of cases diagnosed MEMA I as severe among 599 cases of combined from 5 studies. Out of all cases of combined, 24.1% (95% CI from 7.4 to 40.9%) were reported MEMA I as severe. I^2^ (inconsistency index) was 96.58%.

The whole cohort - Elastic fiber fragmentation and/or loss

Suppl. Fig. 3. The whole cohort – Elastic fibre fragmentation and/or loss

A: The pooled prevalence of cases diagnosed EFF/L as absent among 1084 cases of combined from 8 studies. Out of all cases of combined, 19.4% (95% CI from 4.8 to 33.9%) were reported EFF/L as absent. I^2^ (inconsistency index) was 99.04%. B: The pooled prevalence of cases diagnosed EFF/L as mild among 1084 cases of combined from 8 studies. Out of all cases of combined, 32.5% (95% CI from 18.0 to 47.0%) were reported EFF/L as mild. I^2^ (inconsistency index) was 96.98%. C: The pooled prevalence of cases diagnosed EFF/L as moderate among 1084 cases of combined from 8 studies. Out of all cases of combined, 30.7% (95% CI from 15.5 to 45.9%) were reported EFF/L as moderate. I^2^ (inconsistency index) was 97.59%. D: The pooled prevalence of cases diagnosed EFF/L as severe among 1084 cases of combined from 8 studies. Out of all cases of combined, 16.7% (95% CI from 10.7 to 22.6%) were reported EFF/L as severe. I^2^ (inconsistency index) was 85.04%.

The whole cohort – Elastic fiber thinning

Suppl. Fig. 4. The whole cohort – Elastic fiber thinning

A: The pooled prevalence of cases diagnosed EFT as absent among 600 cases of combined from 6 studies. Out of all cases of combined, 26.5% (95% CI from 10.9 to 42.1%) were reported EFT as absent. I^2^ (inconsistency index) was 98.6%. B: The pooled prevalence of cases diagnosed EFT as mild among 600 cases of combined from 6 studies. Out of all cases of combined, 30.8% (95% CI from 16.2 to 45.5%) were reported EFT as mild. I^2^ (inconsistency index) was 94.65%. C: The pooled prevalence of cases diagnosed EFT as moderate among 600 cases of combined from 6 studies. Out of all cases of combined, 34.7% (95% CI from 16.1 to 53.3%) were reported EFT as moderate. I^2^ (inconsistency index) was 96.91%. D: The pooled prevalence of cases diagnosed EFT as severe among 600 cases of combined from 6 studies. Out of all cases of combined, 6.1% (95% CI from 2.2 to 10.0%) were reported EFT as severe. I^2^ (inconsistency index) was 76.14%.

The whole cohort – Elastic fiber disorganization

Suppl. Fig. 5. The whole cohort – Elastic fiber disorganization

A: The pooled prevalence of cases diagnosed EFD as absent among 345 cases of combined from 5 studies. Out of all cases of combined, 30.5% (95% CI from -0.2 to 61.2%) were reported EFD as absent. I^2^ (inconsistency index) was 99.67%. B: The pooled prevalence of cases diagnosed EFD as focal among 345 cases of combined from 5 studies. Out of all cases of combined, 12.3% (95% CI from 5.1 to 19.4%) were reported EFD as focal. I^2^ (inconsistency index) was 91.26%. C: The pooled prevalence of cases diagnosed EFD as multifocal among 345 cases of combined from 5 studies. Out of all cases of combined, 23.2% (95% CI from 8.9 to 37.5%) were reported EFD as multifocal. I^2^ (inconsistency index) was 95.08%. D: The pooled prevalence of cases diagnosed EFD as extensive among 345 cases of combined from 5 studies. Out of all cases of combined, 6.9% (95% CI from 1.8 to 11.9%) were reported EFD as extensive. I^2^ (inconsistency index) was 89.66%.

The whole cohort – Smooth muscle cell nuclei loss

Suppl. Fig. 6. The whole cohort – Smooth muscle cell nuclei loss

A: The pooled prevalence of cases diagnosed SMCNL as absent among 810 cases of combined from 6 studies. Out of all cases of combined, 23.0% (95% CI from -12.9 to 58.8%) were reported SMCNL as absent. I^2^ (inconsistency index) was 99.82%. B: The pooled prevalence of cases diagnosed SMCNL as mild among 810 cases of combined from 6 studies. Out of all cases of combined, 29.3% (95% CI from 10.9 to 47.7%) were reported SMCNL as mild. I^2^ (inconsistency index) was 97.36%. C: The pooled prevalence of cases diagnosed SMCNL as moderate among 810 cases of combined from 6 studies. Out of all cases of combined, 32.5% (95% CI from 11.4 to 53.6%) were reported SMCNL as moderate. I^2^ (inconsistency index) was 98.04%. D: The pooled prevalence of cases diagnosed SMCNL as severe among 810 cases of combined from 6 studies. Out of all cases of combined, 8.3% (95% CI from 3.7 to 12.9%) were reported SMCNL as severe. I^2^ (inconsistency index) was 85.05%.

The whole cohort – Laminar medial collapse

Suppl. Fig. 7. The whole cohort – Laminar medial collapse

A: The pooled prevalence of cases diagnosed LMC as absent among 581 cases of combined from 4 studies. Out of all cases of combined, 57.5% (95% CI from 30.5 to 84.5%) were reported LMC as absent. I^2^ (inconsistency index) was 98.15%. B: The pooled prevalence of cases diagnosed LMC as mild among 581 cases of combined from 4 studies. Out of all cases of combined, 17.6% (95% CI from 6.0 to 29.2%) were reported LMC as mild. I^2^ (inconsistency index) was 94.56%. C: The pooled prevalence of cases diagnosed LMC as moderate among 581 cases of combined from 4 studies. Out of all cases of combined, 17.3% (95% CI from 7.9 to 26.7%) were reported LMC as moderate. I^2^ (inconsistency index) was 89.83%. D: The pooled prevalence of cases diagnosed LMC as severe among 581 cases of combined from 4 studies. Out of all cases of combined, 5.7% (95% CI from 0.5 to 11.0%) were reported LMC as severe. I^2^ (inconsistency index) was 87.56%.

The whole cohort – Intralamellar medial fibrosis

Suppl. Fig. 8. The whole cohort – Intralamellar medial fibrosis

A: The pooled prevalence of cases diagnosed MF I as absent among 473 cases of combined from 3 studies. Out of all cases of combined, 10.6% (95% CI from 6.2 to 15.0%) were reported MF I as absent. I^2^ (inconsistency index) was 55.73%. B: The pooled prevalence of cases diagnosed MF I as mild among 473 cases of combined from 3 studies. Out of all cases of combined, 49.8% (95% CI from 38.4 to 61.1%) were reported MF I as mild. I^2^ (inconsistency index) was 81.84%. C: The pooled prevalence of cases diagnosed MF I as moderate among 473 cases of combined from 3 studies. Out of all cases of combined, 33.1% (95% CI from 25.7 to 40.5%) were reported MF I as moderate. I^2^ (inconsistency index) was 62.61%. D: The pooled prevalence of cases diagnosed MF I as severe among 473 cases of combined from 3 studies. Out of all cases of combined, 6.5% (95% CI from 4.3 to 8.7%) were reported MF I as severe. I^2^ (inconsistency index) was 0%.

The whole cohort – Translamellar medial fibrosis

Suppl. Fig. 9. The whole cohort - Translamellar medial fibrosis

A: The pooled prevalence of cases diagnosed MF T as absent among 473 cases of combined from 3 studies. Out of all cases of combined, 78.0% (95% CI from 63.5 to 92.4%) were reported MF T as absent. I^2^ (inconsistency index) was 93.09%. B: The pooled prevalence of cases diagnosed MF T as mild among 473 cases of combined from 3 studies. Out of all cases of combined, 3.4% (95% CI from 0.0 to 6.8%) were reported MF T as mild. I^2^ (inconsistency index) was 80.62%. C: The pooled prevalence of cases diagnosed MF T as moderate among 473 cases of combined from 3 studies. Out of all cases of combined, 11.9% (95% CI from 2.0 to 21.9%) were reported MF T as moderate. I^2^ (inconsistency index) was 91.16%. D: The pooled prevalence of cases diagnosed MF T as severe among 473 cases of combined from 3 studies. Out of all cases of combined, 5.9% (95% CI from -0.7 to 12.5%) were reported MF T as severe. I^2^ (inconsistency index) was 91.99%.

The whole cohort - Atherosclerosis

Suppl. Fig. 10. The whole cohort - Atherosclerosis

A: The pooled prevalence of cases diagnosed atherosclerosis as absent among 670 cases of combined from 5 studies. Out of all cases of combined, 52.5% (95% CI from 38.6 to 66.5%) were reported atherosclerosis as absent. I^2^ (inconsistency index) was 92.61%. B: The pooled prevalence of cases diagnosed atherosclerosis as mild among 670 cases of combined from 5 studies. Out of all cases of combined, 30.0% (95% CI from 20.1 to 40.0%) were reported atherosclerosis as mild. I^2^ (inconsistency index) was 87.03%. C: The pooled prevalence of cases diagnosed atherosclerosis as moderate among 670 cases of combined from 5 studies. Out of all cases of combined, 12.2% (95% CI from 8.9 to 15.5%) were reported atherosclerosis as moderate. I^2^ (inconsistency index) was 38.33%. D: The pooled prevalence of cases diagnosed atherosclerosis as severe among 670 cases of combined from 5 studies. Out of all cases of combined, 5.6% (95% CI from 2.1 to 9.0%) were reported atherosclerosis as severe. I^2^ (inconsistency index) was 75.36%.

Aneurysm cohort

The aneurysm cohort – Translamellar mucoid extracellular matrix accumulation

Suppl. Fig. 11. The aneurysm cohort – Translamellar extracellular matrix accumulation

A: The pooled prevalence of cases diagnosed MEMA T as absent among 369 cases of aneurysm from 4 studies. Out of all cases of aneurysm, 25.2% (95% CI from 9.6 to 40.9%) were reported MEMA T as absent. I^2^ (inconsistency index) was 90.68%. B: The pooled prevalence of cases diagnosed MEMA T as mild among 369 cases of aneurysm from 4 studies. Out of all cases of aneurysm, 24.9% (95% CI from 9.3 to 40.4%) were reported MEMA T as mild. I^2^ (inconsistency index) was 89.88%. C: The pooled prevalence of cases diagnosed MEMA T as moderate among 369 cases of aneurysm from 4 studies. Out of all cases of aneurysm, 30.4% (95% CI from 14.0 to 46.9%) were reported MEMA T as moderate. I^2^ (inconsistency index) was 89%. D: The pooled prevalence of cases diagnosed MEMA T as severe among 369 cases of aneurysm from 4 studies. Out of all cases of aneurysm, 15.2% (95% CI from 6.9 to 23.4%) were reported MEMA T as severe. I^2^ (inconsistency index) was 72.96%.

The aneurysm cohort – Intralamellar mucoid extracellular matrix accumulation

Suppl. Fig. 12. The aneurysm cohort – Intralamellar mucoid extracellular matrix accumulation

A: The pooled prevalence of cases diagnosed MEMA I as absent among 369 cases of aneurysm from 4 studies. Out of all cases of aneurysm, 11.2% (95% CI from -0.9 to 23.4%) were reported MEMA I as absent. I^2^ (inconsistency index) was 94.88%. B: The pooled prevalence of cases diagnosed MEMA I as mild among 369 cases of aneurysm from 4 studies. Out of all cases of aneurysm, 24.3% (95% CI from 11.1 to 37.5%) were reported MEMA I as mild. I^2^ (inconsistency index) was 86.36%. C: The pooled prevalence of cases diagnosed MEMA I as moderate among 369 cases of aneurysm from 4 studies. Out of all cases of aneurysm, 39.2% (95% CI from 34.2 to 44.1%) were reported MEMA I as moderate. I^2^ (inconsistency index) was 0.0%. D: The pooled prevalence of cases diagnosed MEMA I as severe among 369 cases of aneurysm from 4 studies. Out of all cases of aneurysm, 24.4% (95% CI from 7.5 to 41.3%) were reported MEMA I as severe. I^2^ (inconsistency index) was 89.51%-

The aneurysm cohort – Elastic fiber fragmentation and/or loss

Suppl. Fig. 13. The aneurysm cohort – Elastic fiber fragmentation and/or loss

A: The pooled prevalence of cases diagnosed EFF/L as absent among 369 cases of aneurysm from 4 studies. Out of all cases of aneurysm, 7.6% (95% CI from 1.3 to 13.9%) were reported EFF/L as absent. I^2^ (inconsistency index) was 75.7%. B: The pooled prevalence of cases diagnosed EFF/L as mild among 369 cases of aneurysm from 4 studies. Out of all cases of aneurysm, 44.2% (95% CI from 25.3 to 63.1%) were reported EFF/L as mild. I^2^ (inconsistency index) was 90.93%. C: The pooled prevalence of cases diagnosed EFF/L as moderate among 369 cases of aneurysm from 4 studies. Out of all cases of aneurysm, 29.5% (95% CI from 18.7 to 40.3%) were reported EFF/L as moderate. I^2^ (inconsistency index) was 72.13%. D: The pooled prevalence of cases diagnosed EFF/L as SEVERE among 369 cases of aneurysm from 4 studies. Out of all cases of aneurysm, 15.6% (95% CI from 3.7 to 27.5%) were reported EFF/L as severe. I^2^ (inconsistency index) was 90.22%.

The aneurysm cohort – Elastic fiber thinning

Suppl. Fig. 14. The aneurysm cohort – Elastic fiber thinning

A: The pooled prevalence of cases diagnosed EFT as absent among 313 cases of aneurysm from 3 studies. Out of all cases of aneurysm, 24.3% (95% CI from 10.2 to 38.5%) were reported EFT as absent. I^2^ (inconsistency index) was 78.12%. B: The pooled prevalence of cases diagnosed EFT as mild among 313 cases of aneurysm from 3 studies. Out of all cases of aneurysm, 48.4% (95% CI from 12.9 to 83.9%) were reported EFT as mild. I^2^ (inconsistency index) was 96.7%. C: The pooled prevalence of cases diagnosed EFT as moderate among 313 cases of aneurysm from 3 studies. Out of all cases of aneurysm, 21.6% (95% CI from -4.0 to 47.2%) were reported EFT as moderate. I^2^ (inconsistency index) was 96.66%. D: The pooled prevalence of cases diagnosed EFT as severe among 313 cases of aneurysm from 3 studies. Out of all cases of aneurysm, 7.1% (95% CI from 1.1 to 13.1%) were reported EFT as severe. I^2^ (inconsistency index) was 67.69%.

The aneurysm cohort – Elastic fiber disorganization

Suppl. Fig. 15. The aneurysm cohort – Elastic fiber disorganization

A: The pooled prevalence of cases diagnosed EFD as absent among 58 cases of aneurysm from 2 studies. Out of all cases of aneurysm, 47.5% (95% CI from -47.5 to 135.6%) were reported EFD as absent. I^2^ (inconsistency index) was 99.62%. B: The pooled prevalence of cases diagnosed EFD as focal among 58 cases of aneurysm from 2 studies. Out of all cases of aneurysm, 29.0% (95% CI from -29.0 to 78.5%) were reported EFD as focal. I^2^ (inconsistency index) was 94.17%. C: The pooled prevalence of cases diagnosed EFD as multifocal among 58 cases of aneurysm from 2 studies. Out of all cases of aneurysm, 21.9% (95% CI from -19.1 to 62.9%) were reported EFD as multifocal. I^2^ (inconsistency index) was 91.86%. D: The pooled prevalence of cases diagnosed EFD as extensive among 58 cases of aneurysm from 2 studies. Out of all cases of aneurysm, 1.5% (95% CI from -1.6 to 4.5%) were reported EFD as extensive. I^2^ (inconsistency index) was 0%.

The aneurysm cohort – Smooth muscle cell nuclei loss

Suppl. Fig. 16. The aneurysm cohort – Smooth muscle cell nuclei loss

A: The pooled prevalence of cases diagnosed SMCNL as absent among 96 cases of aneurysm from 2 studies. Out of all cases of aneurysm, 25.6% (95% CI from 9.5 to 41.7%) were reported SMCNL as absent. I^2^ (inconsistency index) was 71.8%. B: The pooled prevalence of cases diagnosed SMCNL as mild among 96 cases of aneurysm from 2 studies. Out of all cases of aneurysm, 41.3% (95% CI from 30.1 to 52.4%) were reported SMCNL as mild. I^2^ (inconsistency index) was 22.45%. C: The pooled prevalence of cases diagnosed SMCNL as moderate among 96 cases of aneurysm from 2 studies. Out of all cases of aneurysm, 24.2% (95% CI from 10.0 to 38.4%) were reported SMCNL as moderate. I^2^ (inconsistency index) was 62.21%. D: The pooled prevalence of cases diagnosed SMCNL as severe among 96 cases of aneurysm from 2 studies. Out of all cases of aneurysm, 7.3% (95% CI from -5.5 to 20.1%) were reported SMCNL as severe. I^2^ (inconsistency index) was 79.95%.

The aneurysm cohort – Laminar medial collapse

Suppl. Fig. 17. The aneurysm cohort – Laminar medial collapse

A: The pooled prevalence of cases diagnosed LMC as absent among 351 cases of aneurysm from 3 studies. Out of all cases of aneurysm, 55.0% (95% CI from 26.4 to 83.7%) were reported LMC as absent. I^2^ (inconsistency index) was 95.61%. B: The pooled prevalence of cases diagnosed LMC as mild among 351 cases of aneurysm from 3 studies. Out of all cases of aneurysm, 19.7% (95% CI from 0.7 to 38.6%) were reported LMC as mild. I^2^ (inconsistency index) was 93.27%. C: The pooled prevalence of cases diagnosed LMC as moderate among 351 cases of aneurysm from 3 studies. Out of all cases of aneurysm, 20.7% (95% CI from 16.5 to 24.9%) were reported LMC as moderate. I^2^ (inconsistency index) was 0%. D: The pooled prevalence of cases diagnosed LMC as severe among 351 cases of aneurysm from 3 studies. Out of all cases of aneurysm, 1.7% (95% CI from -1.0 to 4.4%) were reported LMC as severe. I^2^ (inconsistency index) was 39.59%.

The aneurysm cohort – Intralamellar medial fibrosis

Suppl. Fig. 18. The aneurysm cohort – Intralamellar medial fibrosis

A: The pooled prevalence of cases diagnosed MF I as absent among 295 cases of aneurysm from 2 studies. Out of all cases of aneurysm, 16.0% (95% CI from 9.1 to 22.8%) were reported MF I as absent. I^2^ (inconsistency index) was 31.19%. B: The pooled prevalence of cases diagnosed MF I as mild among 295 cases of aneurysm from 2 studies. Out of all cases of aneurysm, 45.9% (95% CI from 32.0 to 59.8%) were reported MF I as mild. I^2^ (inconsistency index) was 66.58%. C: The pooled prevalence of cases diagnosed MF I as moderate among 295 cases of aneurysm from 2 studies. Out of all cases of aneurysm, 29.0% (95% CI from 7.7 to 50.2%) were reported MF I as moderate. I^2^ (inconsistency index) was 90.36%. D: The pooled prevalence of cases diagnosed MF I as severe among 295 cases of aneurysm from 2 studies. Out of all cases of aneurysm, 6.1% (95% CI from 3.3 to 8.8%) were reported MF I as severe. I^2^ (inconsistency index) was 0%.

The aneurysm cohort – Translamellar medial fibrosis

Suppl. Fig. 19. The aneurysm cohort – Intralamellar medial fibrosis

A: The pooled prevalence of cases diagnosed MF T as absent among 295 cases of aneurysm from 2 studies. Out of all cases of aneurysm, 69.4% (95% CI from 56.4 to 82.5%) were reported MF T as absent. I^2^ (inconsistency index) was 71.45%. B: The pooled prevalence of cases diagnosed MF T as mild among 295 cases of aneurysm from 2 studies. Out of all cases of aneurysm, 4.8% (95% CI from -2.2 to 11.7%) were reported MF T as mild. I^2^ (inconsistency index) was 59.98%. C: The pooled prevalence of cases diagnosed MF T as moderate among 295 cases of aneurysm from 2 studies. Out of all cases of aneurysm, 15.7% (95% CI from 6.4 to 24.9%) were reported MF T as moderate. I^2^ (inconsistency index) was 68.93%. D: The pooled prevalence of cases diagnosed MF T as severe among 295 cases of aneurysm from 2 studies. Out of all cases of aneurysm, 8.4% (95% CI from -3.0 to 19.8%) were reported MF T as severe. I^2^ (inconsistency index) was 91.96%.

The aneurysm cohort - Atherosclerosis

Suppl. Fig. 20. The aneurysm cohort - Atherosclerosis

A: The pooled prevalence of cases diagnosed atherosclerosis as absent among 334 cases of aneurysm from 3 studies. Out of all cases of aneurysm, 49.1% (95% CI from 25.0 to 73.1%) were reported atherosclerosis as absent. I^2^ (inconsistency index) was 93.38%. B: The pooled prevalence of cases diagnosed atherosclerosis as mild among 334 cases of aneurysm from 3 studies. Out of all cases of aneurysm, 29.3% (95% CI from 16.3 to 42.3%) were reported atherosclerosis as mild. I^2^ (inconsistency index) was 78.54%. C: The pooled prevalence of cases diagnosed atherosclerosis as moderate among 334 cases of aneurysm from 3 studies. Out of all cases of aneurysm, 10.6% (95% CI from 7.3 to 13.9%) were reported atherosclerosis as moderate. I^2^ (inconsistency index) was 0.0%. D: The pooled prevalence of cases diagnosed atherosclerosis as severe among 334 cases of aneurysm from 3 studies. Out of all cases of aneurysm, 10.1% (95% CI from 0.5 to 19.7%) were reported atherosclerosis as severe. I^2^ (inconsistency index) was 90.38%.

Dissection cohort

The dissection cohort – Translamellar mucoid extracellular matrix accumulation

Suppl. Fig. 21. The dissection cohort – Translamellar mucoid extracellular matrix accumulation

A: The pooled prevalence of cases diagnosed MEMA T as absent among 155 cases of dissection from 3 studies. Out of all cases of dissection, 29.8% (95% CI from -6.5 to 66.1%) were reported MEMA T as absent. I^2^ (inconsistency index) was 97.65%. B: The pooled prevalence of cases diagnosed MEMA T as mild among 155 cases of dissection from 3 studies. Out of all cases of dissection, 4.7% (95% CI from -0.9 to 10.2%) were reported MEMA T as mild. I^2^ (inconsistency index) was 64.62%. C: The pooled prevalence of cases diagnosed MEMA T as moderate among 155 cases of dissection from 3 studies. Out of all cases of dissection, 46.6% (95% CI from 15.2 to 78.1%) were reported MEMA T as moderate. I^2^ (inconsistency index) was 94.97%. D: The pooled prevalence of cases diagnosed MEMA T as severe among 155 cases of dissection from 3 studies. Out of all cases of dissection, 17.7% (95% CI from 11.7 to 23.6%) were reported MEMA T as severe. I^2^ (inconsistency index) was 0%.

The dissection cohort – Intralamellar mucoid extracellular matrix accumulation

Suppl. Fig. 22. The dissection cohort – Intralamellar mucoid extracellular matrix accumulation

A: The pooled prevalence of cases diagnosed MEMA I as absent among 155 cases of dissection from 3 studies. Out of all cases of dissection, 9.3% (95% CI from -1.7 to 20.3%) were reported MEMA I as absent. I^2^ (inconsistency index) was 87.12%. B: The pooled prevalence of cases diagnosed MEMA I as mild among 155 cases of dissection from 3 studies. Out of all cases of dissection, 23.3% (95% CI from -4.6 to 51.3%) were reported MEMA I as mild. I^2^ (inconsistency index) was 96.24%. C: The pooled prevalence of cases diagnosed MEMA I as moderate among 155 cases of dissection from 3 studies. Out of all cases of dissection, 27.1% (95% CI from 9.8 to 44.5%) were reported MEMA I as moderate. I^2^ (inconsistency index) was 85.59%. D: The pooled prevalence of cases diagnosed MEMA I as severe among 155 cases of dissection from 3 studies. Out of all cases of dissection, 38.6% (95% CI from -4.5 to 81.6%) were reported MEMA I as severe. I^2^ (inconsistency index) was 98.13%.

The dissection cohort – Elastic fiber fragmentation and/or loss

Suppl. Fig. 23. The dissection cohort – Elastic fiber fragmentation and/or loss

A: The pooled prevalence of cases diagnosed EFF/L as absent among 264 cases of dissection from 5 studies. Out of all cases of dissection, 10.5% (95% CI from 3.2 to 17.8%) were reported EFF/L as absent. I^2^ (inconsistency index) was 92.41%. B: The pooled prevalence of cases diagnosed EFF/L as mild among 264 cases of dissection from 5 studies. Out of all cases of dissection, 25.3% (95% CI from 20.1 to 30.5%) were reported EFF/L as mild. I^2^ (inconsistency index) was 0%. C: The pooled prevalence of cases diagnosed EFF/L as moderate among 264 cases of dissection from 5 studies. Out of all cases of dissection, 41.3% (95% CI from 19.2 to 63.5%) were reported EFF/L as moderate. I^2^ (inconsistency index) was 94.44%. D: The pooled prevalence of cases diagnosed EFF/L as severe among 264 cases of dissection from 5 studies. Out of all cases of dissection, 18.2% (95% CI from 10.6 to 25.7%) were reported EFF/L as severe. I^2^ (inconsistency index) was 64.53%.

The dissection cohort – Elastic fiber thinning

Suppl. Fig. 24. The dissection cohort – Elastic fiber thinning

A: The pooled prevalence of cases diagnosed EFT as absent among 212 cases of dissection from 4 studies. Out of all cases of dissection, 26.4% (95% CI from 11.0 to 41.8%) were reported EFT as absent. I^2^ (inconsistency index) was 98.1%. B: The pooled prevalence of cases diagnosed EFT as mild among 212 cases of dissection from 4 studies. Out of all cases of dissection, 17.0% (95% CI from 11.8 to 22.2%) were reported EFT as mild. I^2^ (inconsistency index) was 7.01%. C: The pooled prevalence of cases diagnosed EFT as moderate among 212 cases of dissection from 4 studies. Out of all cases of dissection, 45.6% (95% CI from 18.5 to 72.8%) were reported EFT as moderate. I^2^ (inconsistency index) was 95.28%. D: The pooled prevalence of cases diagnosed EFT as severe among 212 cases of dissection from 4 studies. Out of all cases of dissection, 6.0% (95% CI from 1.5 to 10.6%) were reported EFT as severe. I^2^ (inconsistency index) was 52.45%.

The dissection cohort – Elastic fiber disorganization

Suppl. Fig. 25. The dissection cohort – Elastic fiber disorganization

A: The pooled prevalence of cases diagnosed EFD as absent among 212 cases of dissection from 4 studies. Out of all cases of dissection, 48.4% (95% CI from 3.5 to 93.3%) were reported EFD as absent. I^2^ (inconsistency index) was 99.85%. B: The pooled prevalence of cases diagnosed EFD as focal among 212 cases of dissection from 4 studies. Out of all cases of dissection, 9.2% (95% CI from 1.5 to 16.9%) were reported EFD as focal. I^2^ (inconsistency index) was 87.68%. C: The pooled prevalence of cases diagnosed EFD as multifocal among 212 cases of dissection from 4 studies. Out of all cases of dissection, 19.5% (95% CI from 4.2 to 34.7%) were reported EFD as multifocal. I^2^ (inconsistency index) was 94.94%. D: The pooled prevalence of cases diagnosed EFD as extensive among 212 cases of dissection from 4 studies. Out of all cases of dissection, 10.6% (95% CI from 2.7 to 18.5%) were reported EFD as extensive. I^2^ (inconsistency index) was 91.48%.

The dissection cohort – Smooth muscle cell nuclei loss

Suppl. Fig. 26. The dissection cohort – Smooth muscle cell nuclei loss

A: The pooled prevalence of cases diagnosed SMCNL as absent among 263 cases of dissection from 5 studies. Out of all cases of dissection, 3.4% (95% CI from 0.0 to 6.9%) were reported SMCNL as absent. I^2^ (inconsistency index) was 68.2%. B: The pooled prevalence of cases diagnosed SMCNL as mild among 263 cases of dissection from 5 studies. Out of all cases of dissection, 30.8% (95% CI from 17.2 to 44.4%) were reported SMCNL as mild. I^2^ (inconsistency index) was 85.34%. C: The pooled prevalence of cases diagnosed SMCNL as moderate among 263 cases of dissection from 5 studies. Out of all cases of dissection, 44.7% (95% CI from 36.9 to 52.5%) were reported SMCNL as moderate. I^2^ (inconsistency index) was 41.78%. D: The pooled prevalence of cases diagnosed SMCNL as severe among 263 cases of dissection from 5 studies. Out of all cases of dissection, 12.1% (95% CI from 5.1 to 19.0%) were reported SMCNL as severe. I^2^ (inconsistency index) was 72.55%.

The dissection cohort – Laminar medial collapse

Suppl. Fig. 27. The dissection cohort – Laminar medial collapse

A: The pooled prevalence of cases diagnosed LMC as absent among 155 cases of dissection from 3 studies. Out of all cases of dissection, 39.5% (95% CI from 2.5 to 76.5%) were reported LMC as absent. I^2^ (inconsistency index) was 96.95%. B: The pooled prevalence of cases diagnosed LMC as mild among 155 cases of dissection from 3 studies. Out of all cases of dissection, 25.1% (95% CI from 10.4 to 39.9%) were reported LMC as mild. I^2^ (inconsistency index) was 80.36%. C: The pooled prevalence of cases diagnosed LMC as moderate among 155 cases of dissection from 3 studies. Out of all cases of dissection, 19.7% (95% CI from 2.6 to 36.8%) were reported LMC as moderate. I^2^ (inconsistency index) was 89.62%. D: The pooled prevalence of cases diagnosed LMC as severe among 155 cases of dissection from 3 studies. Out of all cases of dissection, 12.6% (95% CI from 4.8 to 20.8%) were reported LMC as severe. I^2^ (inconsistency index) was 56.74%.

The dissection cohort – Intralamellar medial fibrosis

Suppl. Fig. 28. The dissection cohort – Intralamellar medial fibrosis

A: The pooled prevalence of cases diagnosed MF I as absent among 103 cases of dissection from 2 studies. Out of all cases of dissection, 8.2% (95% CI from 2.9 to 13.4%) were reported MF I as absent. I^2^ (inconsistency index) was 0%. B: The pooled prevalence of cases diagnosed MF I as mild among 103 cases of dissection from 2 studies. Out of all cases of dissection, 43.1% (95% CI from 24.0 to 62.1%) were reported MF I as mild. I^2^ (inconsistency index) was 75.64%. C: The pooled prevalence of cases diagnosed MF I as moderate among 103 cases of dissection from 2 studies. Out of all cases of dissection, 35.0 % (95% CI from 18.9 to 51.2%) were reported MF I as moderate. I^2^ (inconsistency index) was 67.83%. D: The pooled prevalence of cases diagnosed MF I as severe among 103 cases of dissection from 2 studies. Out of all cases of dissection, 12.0 % (95% CI from 4.8 to 19.2%) were reported MF I as severe. I^2^ (inconsistency index) was 23.4%.

The dissection cohort – Translamellar medial fibrosis

Suppl. Fig. 29. The dissection cohort – Translamellar medial fibrosis

A: The pooled prevalence of cases diagnosed MF T as absent among 103 cases of dissection from 2 studies. Out of all cases of dissection, 81.6% (95% CI from 61.9 to 102.2%) were reported MF T as absent. I^2^ (inconsistency index) was 85.59%. B: The pooled prevalence of cases diagnosed MF T as mild among 103 cases of dissection from 2 studies. Out of all cases of dissection, 6.8% (95% CI from -6.5 to 20.2%) were reported MF T as mild. I^2^ (inconsistency index) was 85.31%. C: The pooled prevalence of cases diagnosed MF T as moderate among 103 cases of dissection from 2 studies. Out of all cases of dissection, 7.7% (95% CI from 2.6 to 12.9%) were reported MF T as moderate. I^2^ (inconsistency index) was 0%. D: The pooled prevalence of cases diagnosed MF T as severe among 103 cases of dissection from 2 studies. Out of all cases of dissection, 3.0% (95% CI from -0.8 to 6.9%) were reported MF T as severe. I^2^ (inconsistency index) was 21.32%.

The dissection cohort - Atherosclerosis

Suppl. Fig. 30. The dissection cohort - Atherosclerosis

A: The pooled prevalence of cases diagnosed atherosclerosis as absent among 261 cases of dissection from 3 studies. Out of all cases of dissection, 49.6% (95% CI from 31.8 to 67.4%) were reported atherosclerosis as absent. I^2^ (inconsistency index) was 87.21%. B: The pooled prevalence of cases diagnosed atherosclerosis as mild among 261 cases of dissection from 3 studies. Out of all cases of dissection, 31.0% (95% CI from 20.1 to 41.9%) were reported atherosclerosis as mild. I^2^ (inconsistency index) was 69.97%. C: The pooled prevalence of cases diagnosed atherosclerosis as moderate among 261 cases of dissection from 3 studies. Out of all cases of dissection, 14.7% (95% CI from 6.6 to 22.8%) were reported atherosclerosis as moderate. I^2^ (inconsistency index) was 69.41%. D: The pooled prevalence of cases diagnosed atherosclerosis as severe among 261 cases of dissection from 3 studies. Out of all cases of dissection, 3.8% (95% CI from 1.5 to 6.1%) were reported atherosclerosis as severe. I^2^ (inconsistency index) was 0%.

The BAV cohort

The BAV cohort – Translamellar mucoid extracellular matrix accumulation

Suppl. Fig. 31. The BAV cohort – Translamellar mucoid extracellular matrix accumulation

A: The pooled prevalence of cases diagnosed MEMA T as absent among 75 cases of BAV from 3 studies. Out of all cases of BAV, 65.4% (95% CI from 54.6 to 76.2%) were reported MEMA T as absent. I^2^ (inconsistency index) was 0%. B: The pooled prevalence of cases diagnosed MEMA T as mild among 75 cases of BAV from 3 studies. Out of all cases of BAV, 17.5% (95% CI from -2.2 to 37.2%) were reported MEMA T as mild. I^2^ (inconsistency index) was 75.09%. C: The pooled prevalence of cases diagnosed MEMA T as moderate among 75 cases of BAV from 3 studies. Out of all cases of BAV, 7.7% (95% CI from 1.7 to 13.8%) were reported MEMA T as moderate. I^2^ (inconsistency index) was 0%. D: The pooled prevalence of cases diagnosed MEMA T as severe among 75 cases of BAV from 3 studies. Out of all cases of BAV, 9.7% (95% CI from -14.7 to 34.1%) were reported MEMA T as severe. I^2^ (inconsistency index) was 60.01%.

The BAV cohort – Intralamellar mucoid extracellular matrix accumulation

Suppl. Fig. 32. The BAV cohort – Intralamellar mucoid extracellular matrix accumulation

A: The pooled prevalence of cases diagnosed MEMA I as absent among 75 cases of BAV from 2 studies. Out of all cases of BAV, 0.8% (95% CI from -1.2 to 2.8%) were reported MEMA I as absent. I^2^ (inconsistency index) was 0%. B: The pooled prevalence of cases diagnosed MEMA I as mild among 75 cases of BAV from 2 studies. Out of all cases of BAV, 46.4% (95% CI from 23.0 to 69.8%) were reported MEMA I as mild. I^2^ (inconsistency index) was 50.87%. C: The pooled prevalence of cases diagnosed MEMA I as moderate among 75 cases of BAV from 2 studies. Out of all cases of BAV, 45.3% (95% CI from 34.1 to 56.6%) were reported MEMA I as moderate. I^2^ (inconsistency index) was 0%. D: The pooled prevalence of cases diagnosed MEMA I as severe among 75 cases of BAV from 2 studies. Out of all cases of BAV, 9.4% (95% CI from -15.9 to 34.7%) were reported MEMA I as moderate. I^2^ (inconsistency index) was 62.27%.

The BAV cohort – Elastic fiber fragmentation and/or loss

Suppl. Fig. 33. The BAV cohort – Elastic fiber fragmentation and/or loss

A: The pooled prevalence of cases diagnosed EFF/L as absent among 284 cases of BAV from 3 studies. Out of all cases of BAV, 48.9% (95% CI from 12.2 to 85.7%) were reported EFF/L as absent. I^2^ (inconsistency index) was 96.53%. B: The pooled prevalence of cases diagnosed EFF/L as mild among 284 cases of BAV from 3 studies. Out of all cases of BAV, 24.5% (95% CI from -8.4 to 57.5%) were reported EFF/L as mild. I^2^ (inconsistency index) was 95.77%. C: The pooled prevalence of cases diagnosed EFF/L as moderate among 284 cases of BAV from 3 studies. Out of all cases of BAV, 7.0% (95% CI from -1.3 to 15.3%) were reported EFF/L as moderate. I^2^ (inconsistency index) was 69.16%. D: The pooled prevalence of cases diagnosed EFF/L as severe among 284 cases of BAV from 3 studies. Out of all cases of BAV, 7.9% (95% CI from -1.7 to 17.6%) were reported EFF/L as severe. I^2^ (inconsistency index) was 87.74%.

The BAV cohort – Elastic fiber thinning

Suppl. Fig. 34. The BAV cohort – Elastic fiber thinning

A: The pooled prevalence of cases diagnosed EFT as absent among 75 cases of BAV from 2 studies. Out of all cases of BAV, 74.0% (95% CI from 64.2 to 83.9%) were reported EFT as absent. I^2^ (inconsistency index) was 0%. B: The pooled prevalence of cases diagnosed EFT as mild among 75 cases of BAV from 2 studies. Out of all cases of BAV, 16.2% (95% CI from -0.5 to 33.0%) were reported EFT as mild. I^2^ (inconsistency index) was 66.61%. C: The pooled prevalence of cases diagnosed EFT as moderate among 75 cases of BAV from 2 studies. Out of all cases of BAV, 4.7% (95% CI from -0.0 to 9.5%) were reported EFT as moderate. I^2^ (inconsistency index) was 0%. D: The pooled prevalence of cases diagnosed EFT as severe among 75 cases of BAV from 2 studies. Out of all cases of BAV, 0.8% (95% CI from -1.2 to 2.8%) were reported EFT as severe. I^2^ (inconsistency index) was 0%.

The BAV cohort – Smooth muscle cell nuclei loss

Suppl. Fig. 35. The BAV cohort – Smooth muscle cell nuclei loss

A: The pooled prevalence of cases diagnosed SMCNL as absent among 284 cases of BAV from 3 studies. Out of all cases of BAV, 37.7% (95% CI from -39.6 to 114.9%) were reported SMCNL as absent. I^2^ (inconsistency index) was 99.95%. B: The pooled prevalence of cases diagnosed SMCNL as mild among 284 cases of BAV from 3 studies. Out of all cases of BAV, 45.5% (95% CI from -6.8 to 97.7%) were reported SMCNL as mild. I^2^ (inconsistency index) was 98.52%. C: The pooled prevalence of cases diagnosed SMCNL as moderate among 284 cases of BAV from 3 studies. Out of all cases of BAV, 12.3% (95% CI from -8.7 to 33.3%) were reported SMCNL as moderate. I^2^ (inconsistency index) was 93.4%. D: The pooled prevalence of cases diagnosed SMCNL as severe among 284 cases of BAV from 3 studies. Out of all cases of BAV, 1.0% (95% CI from -0.9 to 2.9%) were reported SMCNL as severe. I^2^ (inconsistency index) was 18%.

The BAV cohort – Laminar medial collapse

Suppl. Fig. 36. The BAV cohort – Laminar medial collapse

A: The pooled prevalence of cases diagnosed LMC as absent among 75 cases of BAV from 2 studies. Out of all cases of BAV, 96.8% (95% CI from 92.8 to 100.8%) were reported LMC as absent. I^2^ (inconsistency index) was 0%. B: The pooled prevalence of cases diagnosed LMC as mild among 75 cases of BAV from 2 studies. Out of all cases of BAV, 0.8% (95% CI from -1.2 to 2.8%) were reported LMC as mild. I^2^ (inconsistency index) was 0%. C: The pooled prevalence of cases diagnosed LMC as moderate among 75 cases of BAV from 2 studies. Out of all cases of BAV, 3.1% (95% CI from -0.8 to 7.0%) were reported LMC as moderate. I^2^ (inconsistency index) was 0%. D: The pooled prevalence of cases diagnosed LMC as severe among 75 cases of BAV from 2 studies. Out of all cases of BAV, 1.1% (95% CI from -3.8 to 5.4%) were reported LMC as severe. I^2^ (inconsistency index) was 4.31%.

The BAV cohort – Intralamellar medial fibrosis

Suppl. Fig. 37. The BAV cohort – Intralamellar medial fibrosis

A: The pooled prevalence of cases diagnosed MF I as absent among 76 cases of BAV from 2 studies. Out of all cases of BAV, 0.8% (95% CI from -1.2 to 2.7%) were reported MF I as absent. I^2^ (inconsistency index) was 0%. B: The pooled prevalence of cases diagnosed MF I as mild among 76 cases of BAV from 2 studies. Out of all cases of BAV, 71.1% (95% CI from 60.9 to 81.2%) were reported MF I as mild. I^2^ (inconsistency index) was 0%. C: The pooled prevalence of cases diagnosed MF I as moderate among 76 cases of BAV from 2 studies. Out of all cases of BAV, 27.6% (95% CI from 17.6 to 37.7%) were reported MF I as moderate. I^2^ (inconsistency index) was 0%. D: The pooled prevalence of cases diagnosed MF I as severe among 76 cases of BAV from 2 studies. Out of all cases of BAV, 0.8% (95% CI from -1.2 to 2.8%) were reported MF I as severe. I^2^ (inconsistency index) was 0%.

The BAV cohort – Translamellar medial fibrosis

Suppl. Fig. 38. The BAV cohort – Translamellar medial fibrosis

A: The pooled prevalence of cases diagnosed MF T as absent among 75 cases of BAV from 2 studies. Out of all cases of BAV, 80.8% (95% CI from 44.2 to 117.5%) were reported MF T as absent. I^2^ (inconsistency index) was 75.91%. B: The pooled prevalence of cases diagnosed MF T as mild among 75 cases of BAV from 2 studies. Out of all cases of BAV, 4.6% (95% CI from -0.1 to 9.2%) were reported MF T as mild. I^2^ (inconsistency index) was 0%. C: The pooled prevalence of cases diagnosed MF T as moderate among 75 cases of BAV from 2 studies. Out of all cases of BAV, 17.7% (95% CI from -22.8 to 58.1%) were reported MF T as moderate. I^2^ (inconsistency index) was 80.23%. D: The pooled prevalence of cases diagnosed MF T as severe among 75 cases of BAV from 2 studies. Out of all cases of BAV, 0.8% (95% CI from -1.2 to 2.8%) were reported MF T as severe. I^2^ (inconsistency index) was 0%.

The BAV cohort - Atherosclerosis

Suppl. Fig. 39. The BAV cohort - Atherosclerosis

A: The pooled prevalence of cases diagnosed atherosclerosis as absent among 76 cases of BAV from 2 studies. Out of all cases of BAV, 47.7% (95% CI from 8.1 to 87.4%) reported atherosclerosis as absent. I^2^ (inconsistency index) was 80.83%. B: The pooled prevalence of cases diagnosed atherosclerosis as mild among 76 cases of BAV from 2 studies. Out of all cases of BAV, 36.4% (95% CI from -3.4 to 76.2%) were reported atherosclerosis as mild. I^2^ (inconsistency index) was 87.33%. C: The pooled prevalence of cases diagnosed atherosclerosis as moderate among 76 cases of BAV from 2 studies. Out of all cases of BAV, 11.8% (95% CI from 4.6 to 19.1%) were reported atherosclerosis as moderate. I^2^ (inconsistency index) was 0%. D: The pooled prevalence of cases diagnosed atherosclerosis as severe among 76 cases of BAV from 2 studies. Out of all cases of BAV, 1.6% (95% CI from -1.2 to 4.4%) were reported atherosclerosis as severe. I^2^ (inconsistency index) was 0%.
